# Substrate-Driven Stabilization of Perpendicular Magnetic Anisotropy and Near-Room-Temperature Ferromagnetism in Cr-Rich Cr_1+δ_Te_2_ Films

**DOI:** 10.1021/acs.jpcc.5c02927

**Published:** 2025-09-05

**Authors:** Akylas Lintzeris, Polychronis Tsipas, Shanshan Guo, Panagiotis Pappas, Elli Georgopoulou−Kotsaki, Ilya Kostanovski, Claudia Felser, Edouard Lesne, Hanako Okuno, Athanasios Dimoulas

**Affiliations:** † Institute of Nanoscience and Nanotechnology, 54572 National Center for Scientific Research “Demokritos”, 15310 Athens, Greece; ‡ School of Applied Mathematical and Physical Sciences, National Technical University of Athens, 15780 Athens, Greece; § Max Planck Institute for Chemical Physics of Solids, 01187 Dresden, Germany; ∥ 28286 Max Planck Institute of Microstructure Physics, 06120 Halle, Saale, Germany; ⊥ CEA, IRIG-MEM, Université Grenoble Alpes, 38000 Grenoble, France

## Abstract

Thin films of Cr-rich self-intercalated Cr_1+δ_Te_2_ two-dimensional (2D) ferromagnet typically exhibit high Curie critical temperature (*T*_C_) with in-plane magnetic anisotropy. In this work, we show that high quality Cr-rich (δ = 0.76) Cr_1+δ_Te_2_ films grown on Si/InAs substrates by molecular beam epitaxy, at high temperature, exhibit a new magnetic phase combining perpendicular magnetic anisotropy (PMA) with a near-room-temperature Curie temperature (*T*_C_ ≈ 260 K), albeit with slightly reduced saturation magnetization, as evidenced by SQUID and magneto-optical Kerr effect magnetometry. The new phase manifests itself by a characteristic Moiré pattern as revealed by scanning tunneling microscopy and is associated with a contraction of the *c*-axis by 0.08 Å, as evidenced by X-ray diffraction, which are both attributed to indium diffusion and segregation at the surface. First-principles calculations indicate a noncollinear magnetic moment configuration that can be attributed to nearest-neighbor interlayer antiferromagnetic exchange interaction. This configuration shifts toward increased collinearity as the *c*-axis contracts and is attributed to the strengthening of the ferromagnetic exchange interaction which favors PMA. This work highlights that it is possible to influence the magnetic state of 2D chromium telluride ferromagnets, using suitable substrates and growth parameters to obtain PMA at nearly room temperature, which is desirable for future spintronics and prospective magnetic memories applications.

## Graphical abstract

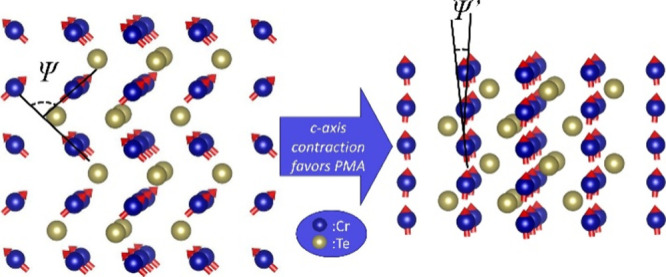

## Introduction

Two-dimensional (2D) van der Waals (vdW)^1^ magnetic materials, which host a plethora of magnetic groundstates, have attracted significant interest in the field of spintronics.^2^ Initially, intrinsic, long-range magnetic order was confirmed to a range of 2D ferromagnets with Curie temperatures (*T*_C_) significantly lower than room temperature (RT).^3–5^ Recent advances have revealed that the class of 2D ferromagnetic (FM) materials contains a large number of FMs that either exhibit *T*_C_ values above RT^6–8^ or has a *T*_C_ that can be manipulated in several ways such as electrostatic ionic gating,^9^ interfacing with other materials like Bi_2_Te_3_ ^10,11^ and ultrafast light-induced carrier doping.^12^ Among the class of 2D FM materials, Cr_1+δ_Te_2_ (δ varying from 0 to 1) compounds have come under notable attention since seminal works have provided evidence for intrinsic RT ferromagnetism down to a few atomic layers.^8,13–15^ Depending on the thickness and composition, a number of Cr_1+δ_Te_2_ compounds have shown perpendicular magnetic anisotropy (PMA), which is highly desirable for a number of spintronic and magnetic memory devices. Promising results on the CrTe_2_ phase^16,17^ motivated a systematic investigation of structural and magnetic properties of Cr_1+δ_Te_2_ compounds as a function of δ.^18–20^ The structure and composition of Cr_1+δ_Te_2_ compounds are complex due to the self-intercalated Cr atoms between 1T-CrTe_2_ layers,^21^ that give rise to different stable stoichiometries determined by the factor δ, which represents the portion of the intercalants. Stable compositions of Cr_1+δ_Te_2_ have been synthesized and studied, including CrTe_2_, Cr_5_Te_8_, Cr_2_Te_3_, and Cr_3_Te_4_.^16,18,22,23^ Different phases and stoichiometries exhibit dissimilar magnetic properties, varying from phases with strong PMA^10,16,24,25^ to others where the easy magnetization axis lies in-plane.^22,26,27^ The Curie temperature, which is affected by a number of factors, including, e.g., stoichiometry and thickness,^17,27–29^ ranges from 140 K^30^ to temperatures significantly above RT.^16^ Moreover, Cr_1+δ_Te_2_ 2D FM has garnered significant attention due to its potential for high-performance nanoelectronics and skyrmion-based applications, driven by phenomena such as the large anomalous Hall effect (AHE)^31–33^ and topologically protected spin textures such as skyrmions.^10,34,35,23^ Cr_1+δ_Te_2_ has been successfully synthesized with all the prevalent material growth techniques, such as molecular beam epitaxy (MBE),^27,36^ chemical vapor deposition,^23,26^ and pulsed laser deposition.^37^

The main trend in the magnetic properties in relation to the composition is as follows:^10,27^ for low growth temperature (*T*_g_ < 250 °C), the compound is less Cr-rich with composition matching the Cr_2_Te_3_ phase due to limited Cr self-intercalation and features a low *T*_C_ (∼160 K) with PMA. At elevated *T*_g_ (>400 °C), Cr self-intercalation increases, yielding Cr-rich phases that show *T*_C_ > 300 K and in-plane magnetic anisotropy. Furthermore, compounds that display higher *T*_C_ values also exhibit a stronger tendency toward in-plane magnetic anisotropy, at odds with the desired combined PMA and *T*_C_ > RT. Thus, achieving Cr_1+δ_Te_2_ synthesis with near or higher than RT *T*_C_—while maintaining strong PMA—remains an important target and a challenging task.

Notably, controlling the optimum composition (δ) by adjusting the growth temperature or via post-growth annealing procedures is famously difficult. Slight changes in the synthesis conditions result in different values of δ and, consequently, different chromium telluride phases.^27^

In the present work, we have systematically studied the influence of the substrate on the controllable growth of Cr_1+δ_Te_2_ phase with the desired high *T*_C_ and PMA characteristics. We found that Cr_1+δ_Te_2_ grown on a variety of substrates at a high growth temperature (*T*_g_) of 425 °C show ferromagnetism with *T*_C_ > 300 K and in-plane magnetic anisotropy. A notable exception occurs when we use InAs as a substrate. In this case, the layer displays a characteristic Moiré pattern revealed by scanning tunneling microscopy (STM) and exhibits ferromagnetism with characteristic PMA and *T*_C_ around 260 K. Combining near room temperature ferromagnetism with PMA in Cr_1+δ_Te_2_ is rare and creates prospects for applications in spintronics. It is anticipated that due to the high *T*_g_, indium diffuses during the growth and further segregates at the surface. This process alters growth kinetics on the surface and inside the film, resulting in Cr_1+δ_Te_2_ phases with favored PMA and enhanced *T*_C_. In contrast, growing Cr_1+δ_Te_2_ on InAs but at low *T*_g_ (∼225 °C) yields PMA but with a lower *T*_C_ (∼160 K), which is expected for Cr_2_Te_3_ phases based on literature reports.^28,38^ This supports our assumption about indium diffusion, which occurs only at high *T*_g_. Indium diffusion is also detected by X-ray photoemission spectroscopy (XPS) in relatively thick layers, and its segregation on the topmost Cr_1+δ_Te_2_ surface layers is further supported by high-resolution transmission electron microscopy (HRTEM) and is compatible with the observation of a Moiré pattern in STM. Our work suggests a new way to influence the growth of desired Cr_1+δ_Te_2_ phases by using suitable substrates that interact with the grown layers.

## Methods

### Molecular Beam Epitaxy Growth

Chromium telluride thin films are grown by MBE on Si(111)/InAs(111), Si(111)/AlN, sapphire/h-BN, sapphire/graphene and sapphire(0001)/WS_2_(001) substrates in a wide range of growth temperature, from 225 °C to 475 °C. Cr and Te are evaporated from an e-beam evaporator and a thermal cracker cell, respectively. During the growth, the pressure of the ultrahigh vacuum (UHV) chamber is kept at 10^–8^ Torr. The deposition rate for Cr is 0.06 Å/s and Te is in overpressure with Te/Cr ratio of 15/1 Å/s. Samples of different thicknesses were synthesized, ranging from 2 nm to 16 nm. All samples are in-situ capped by either Al or W, to protect them from oxidization.

### Structural and Chemical Characterization

XPS was carried out to analyze the chemical state of the surface of the samples and to detect indium diffusion from the substrate. The measurements were performed using a Mg Kα X-ray source with a photon energy of 1253.64 eV, and the spectra were collected with a PHOIBOS 100 (SPECS) hemispherical analyzer. The surface of the samples was monitored in situ via reflection high-energy electron diffraction (RHEED), while the Moiré pattern observed in high-*T*_g_ samples grown on InAs was characterized using STM. To investigate the structural and compositional properties of Cr_1+δ_Te_2_ with δ = 0.76 on InAs, cross-sectional TEM combined with energy-dispersive X-ray spectroscopy (EDX) was employed. Additionally, X-ray diffraction (XRD) analysis was carried out in Bragg–Brentano geometry, using a Siemens D5000 diffractometer with Cu Kα radiation over the 2θ range of 10°–90°, with a step size of 0.03° and an integration time of 8 s per step, in order to evaluate *c*-axis lattice contraction. The stoichiometry of four representative samples, presented in the Supporting Information, was determined by Rutherford backscattering spectrometry (RBS).

### Magnetic Properties Characterization

Magnetization measurements were carried out with a Quantum Design MPMS3 SQUID-VSM magnetometer, providing *M*–*H* and *M*–*T* curves for selected samples. In addition, magneto-optical Kerr effect (MOKE) microscopy and magnetometry were performed using a system from Evico Magnetics, equipped with a continuous-flow liquid nitrogen optical cryostat, enabling measurements in the temperature range of 77–320 K.

### First-Principles Calculations

First-principles calculations were performed using VASP^39,40^ to explore the magnetic configuration as a function of the *c*-axis value. We used as the pseudopotential an all-electron projector-augmented wave (PAW) potential.^41^ As for the exchange-correlation functional, the generalized-gradient approximation (GGA) of Perdew–Burke–Ernzerhof (PBE)^42^ was chosen. The kinetic energy cutoff of the plane-wave basis set was fixed to 500 eV and the energy convergence criterion was set to 10^–7^ eV. A dense *k*-point sampling of the Brillouin zone was implemented by the Monkhorst–Pack scheme with an 18 × 18 × 8 grid.

## Experimental Results

### Indium Incorporation in the Film

Most of the data presented below are collected from the chromium telluride films grown by MBE on InAs(111) templates because they show a distinct Moiré superstructure on the surface associated with near RT ferromagnetism and PMA. Figure 1 shows in-situ XPS of three thicknesses of Cr_1+δ_Te_2_ films (16, 36, and 104 nm) grown on InAs(111) templates under the same *T*_g_ (425 °C). For such thick samples, spectroscopic signatures of indium from the InAs substrate should not be visible, due to the probing depth of about 5 nm. The clear observation of the indium 3d_3/2_ and 3d_5/2_ peaks (Figure 1) likely indicates the presence of In on the surface of the film, promoted by diffusion of In from the InAs substrate during the growth at an elevated temperature. Moreover, as the thickness of the sample increases, the indium signal becomes stronger, indicating that, for longer growth durations, more indium is incorporated into the topmost layers of the film. On the other hand, the intensity from tellurium peaks (see inset of Figure 1) remain essentially unaffected. Additional TEM investigations presented hereafter help rule out the scenario, whereby the In would be evenly distributed throughout the films’ thickness.

**Figure 1:**
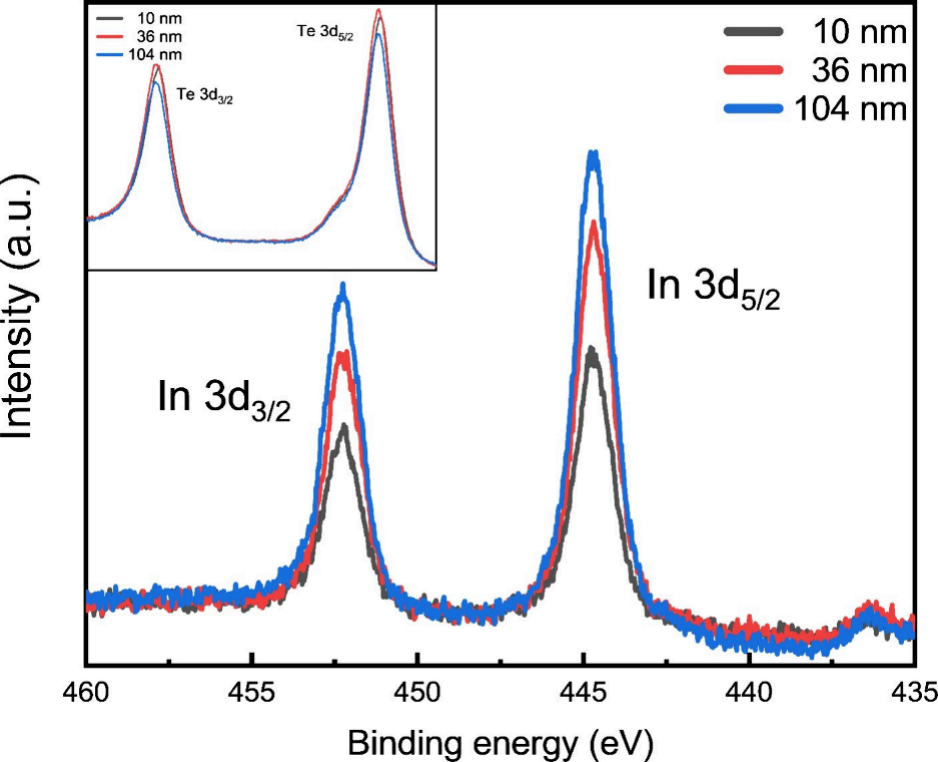
XPS spectrum of In 3d and Te 3d (inset) core levels for Cr_1+δ_Te_2_ films of various thicknesses grown at 425 °C. The tellurium peaks’ intensities do not change significantly with the thickness of the Cr_1+δ_Te_2_ films, while the indium peaks’ intensities grow stronger for thicker samples.

### TEM Analysis

Figure 2 presents TEM cross-section of W-capped 10-nm-thick Cr_1+δ_Te_2_ films on a InAs(111) template (Figure 2a) and the corresponding depth-resolved intensity profile (Figure 2b). Cross-sectional TEM combined with element-specific EDX spectroscopy imaging is presented in Figures 2c–g. The vdW gap is clearly visible in Figure 2a and further revealed in Figure 2b, although it is difficult to detect intercalated Cr in the vdW gap. From the spatially resolved element analysis, it can be revealed that indium is predominantly accumulated at the Cr_1+δ_Te_2_/W interface. The small traces of indium inside the film in Figure 2g are at the noise level. In Figures 2d–g, we observe a Cr-deficient layer below the W capping layer, which coincides with the In-rich topmost layers of the film. Concomitantly, the Te signal does not noticeably decrease. This indicates the possible formation of an indium tellurium layer at the Cr_1+δ_Te_2_/W interface. The presence of indium at the surface agrees with the XPS data in Figure 1 and helps interpret the Moiré pattern obtained by STM as discussed below.

**Figure 2:**
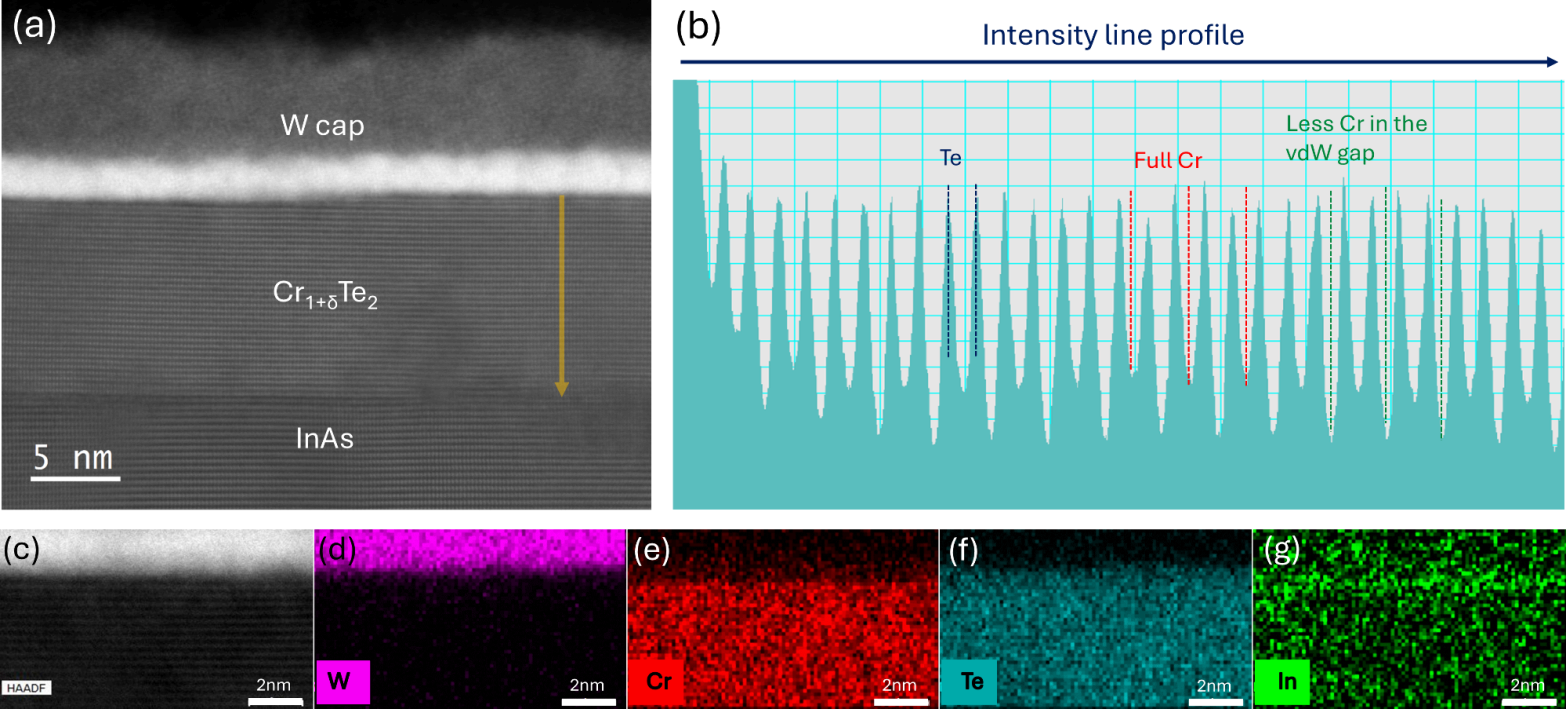
Transmission electron microscopy measurements and analysis of a 10-nm-thick Cr_1+δ_Te_2_ sample capped with W. (a) Cross-sectional image showing polycrystalline W capping layer and lattice fringes of the crystalline InAs substrate and Cr_1+δ_Te_2_ film. (b) CrTe_2_ layers and vdW gaps are clearly observed in the intensity profile of the Cr_1+δ_Te_2_ layer. (c–g) EDX data present the elements imaging and indicate tellurium (panel (f)) and indium segregation (panel (g)) at the Cr_1+δ_Te_2_/W interface.

### Reflection High-Energy Electron Diffraction (RHEED) and STM Analysis

Figure 3 shows in-situ RHEED (see Figures 3a and 3d) and STM images (Figures 3b, 3c, 3e, and 3f) of the surface of the chromium telluride films grown on InAs(111) templates at elevated temperatures. The film grown at 400 °C (Figures 3a–c) consists of two phases spatially separated as indicated by areas A and M. Area M is clearly distinguished due to a characteristic periodic Moiré pattern. From the RHEED pattern (Figure 3a), it is seen that the two areas have different lattice constants of *a*_A_ = 3.87 Å (area A) and *a*_M_ = 4.364 Å (area M). At higher growth temperature of 425 °C, the whole layer consists of one phase (Figure 3d) exhibiting the Moiré pattern (Figure 3e). The atomic resolution image of area A in the inset of Figure 3c shows 2 × 1 rotated domains compatible with the ×2 superstructure in RHEED (Figure 3a). The corresponding atomic resolution image of area M in Figure 3f shows a Moiré pattern rotationally aligned with the lattice (φ = 0) and with Moiré period λ_Μ_ ≈ 10*a*_M_. It is speculated that in areas M, a single layer of In-containing material lies on top of a Cr_1+δ_Te_2_ layer (also evidenced from Figure 2g) yielding the observed Moiré interference due to a lattice mismatch ε between the two layers. It should be noted that the presence of In on the Cr_1+δ_Te_2_ surface has been detected by TEM (see Figure 2). Applying the known formula,^43^

1 $$\lambda_{M}\left(\varphi ,\varepsilon ,a_{Μ}\right)=a_{M}\left(1+\varepsilon \right){\left[2\left(1+\varepsilon \right)\left(1-\cos\;\varphi \right)+\varepsilon^{2}\right]}^{-1/2}$$

and setting φ = 0, we solve eq 1 for ε to obtain ε = [(*λ*_Μ_/(α_Μ_) – 1]^−1^ = 13.1%. This value agrees well with the value of 12.8% estimated from the lattice mismatch (*a*_M_ – *a*_Α_)/*a*_A_ between areas A and M. Notably, the value of *a*_M_ is close to the value of the In_2_Te_3_ lattice parameter of 4.36 Å,^44^ suggesting that the top In-containing layer is In_2_Te_3_. It is anticipated that, at elevated temperature (*T*_*g*_ > 400 °C), indium diffuses through the grown layer, floating at the surface and acting as a surfactant, reducing the surface energy during growth and leading to layer-by-layer (Frank–Van der Merwe) deposition.^45^ This results in the smooth growth of the layer atop the M areas that display a surface root-mean-square roughness of less than 0.25 nm acquired from the terrace presented in Figure 3e. It should be noted that Cr_1+δ_Te_2_ grown on InAs at a lower temperature of 225 °C, shows a different surface (see Figure S2), whereby the Moiré structure is totally absent and the film shows a faint 2 × 2 superstructure reminiscent of a phase with low Cr content, and corresponding to δ ≤ 0.33 and a low *T*_C_ (∼160 K).^10^

**Figure 3:**
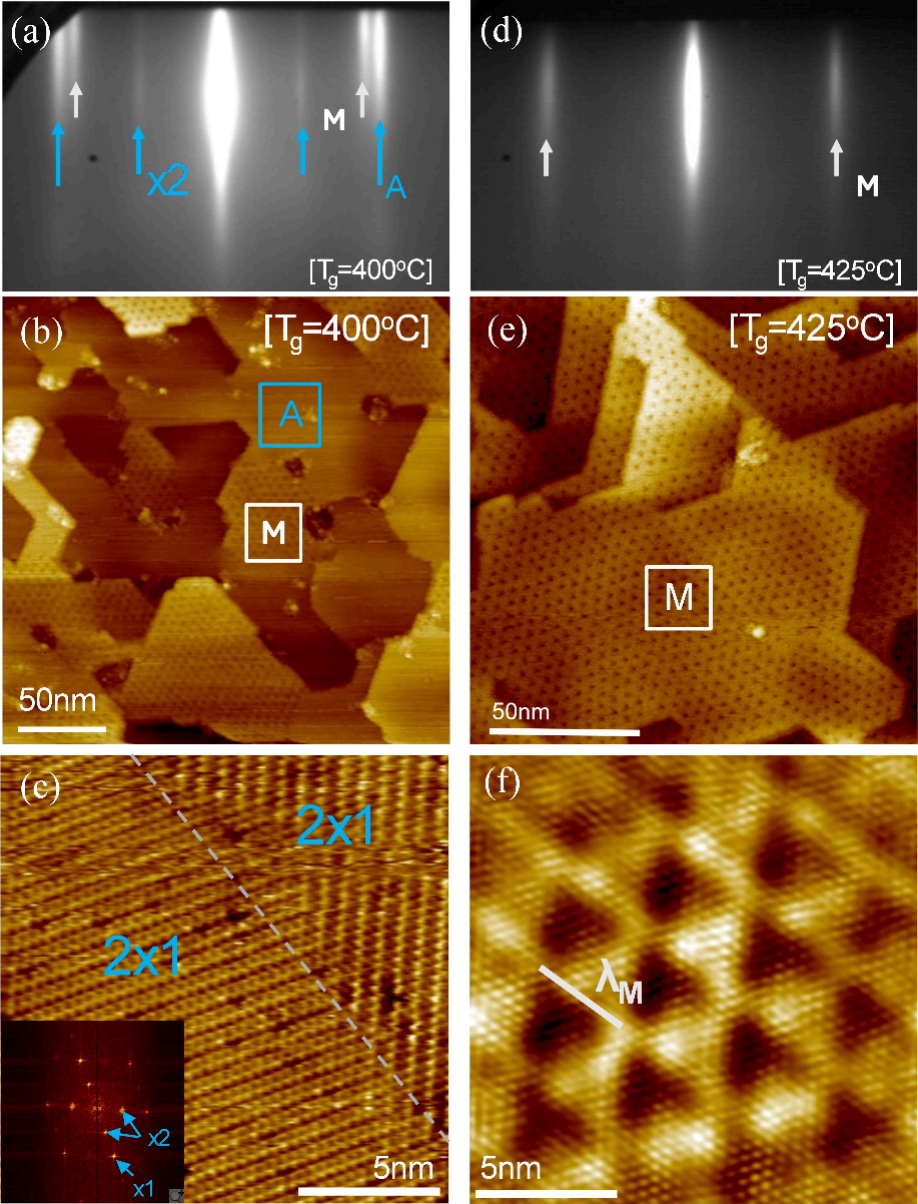
In-situ surface characterization by RHEED (panels (a) and (d)) and STM (panels (b), (c), (e), (f)) of the samples grown on InAs. (a) Sample grown at an intermediate temperature of 400 °C. Two phases with lattice constants *a* = 3.87 Å (phase A, blue arrows) and *a* = 4.364 Å (phase M, white arrows) coexist. A faint ×2 (blue arrows) surface reconstruction is also present associated with phase A. (b) STM data of the sample grown at the intermediate *T*_g_ = 400 °C, shows an inhomogeneous surface where both phases A and M coexist next to each other. Phase M is clearly distinguished from phase A by a clear trigonal Moiré pattern. (c) Phase A exhibits a 2 × 1 surface reconstruction with rotated domains clearly observed both in the real image and corresponding fast Fourier transform. (d) High-*T*_g_ grown sample shows the presence of phase M only, with an estimated lattice constant of *a* = 4.364 Å. (inset). This explains the ×2 reconstruction present in the RHEED pattern in panel (a). (e) The sample grown at *T*_g_ = 425 °C exhibits a homogeneous surface where only phase M with the characteristic Moiré pattern is present. (f) High-resolution image shows the Moiré pattern in detail, with a period λ_m_ = 9*a*. The film surface shows a terrace-like structure with a very small amount of roughness in each terrace.

Phase A with lattice constant *a*_A_ = 3.87 Å, which is observed next to the Moiré phase, is identified as chromium telluride phase based on the available data.^27,46^ Chromium telluride in-plane lattice constant exhibits small variations with the amount of intercalated Cr, the growth conditions and the choice of substrate.

### X-ray Diffractometry and Rutherford Backscattering Analysis

The composition of the films was analyzed using RBS (see the Supporting Information). The RBS measurements reveal that the layer grown on InAs at high *T*_g_ has the largest Cr content. In Cr_1+δ_Te_2_ compounds, the intercalation of Cr atoms between the vdW gaps typically leads to a slight expansion of the *c*-axis lattice parameter,^27,46^ as additional atoms increase the interlayer spacing. It is thus expected that the *c*-axis would have the largest value, compared to other samples with lower Cr content. However, X-ray diffraction in Figure 4 (and Figure S3) reveals that the film grown at high *T*_g_ on InAs substrates (sample A, green line) has a *c*-axis lattice parameter of 6.07 Å, which is reduced, compared to other Cr_1+δ_Te_2_ layers with similar composition grown on different substrates (e.g., on sapphire; sample B, red line) or to layers grown at lower *T*_g_ with lower Cr content (sample C, blue line), both having *c* ≈ 6.15 Å (Figure 4). The unexpected contraction of the *c*-axis is attributed to the influence of In diffusion in the case of sample A and the formation of an atomically thin indium telluride layer at the Cr_1+δ_Te_2_/capping interface, as revealed by TEM (Figure 2). Although indium is a larger ion than chromium, the observed contraction of the *c*-axis in Cr_1+δ_Te_2_ films grown on InAs at high growth temperatures cannot be attributed to direct substitutional incorporation of indium into the lattice. We propose instead that indium may act as a surfactant during growth,^47,48^ modifying surface kinetics and, by suppressing lattice relaxation, it builds biaxial tensile strain. As a result, a contraction is observed by XRD in the out-of-plane lattice parameter along the *c*-axis. The contraction of *c* may have an influence on the magnetic properties of the samples grown on InAs at high *T*_g_, as discussed below.

**Figure 4:**
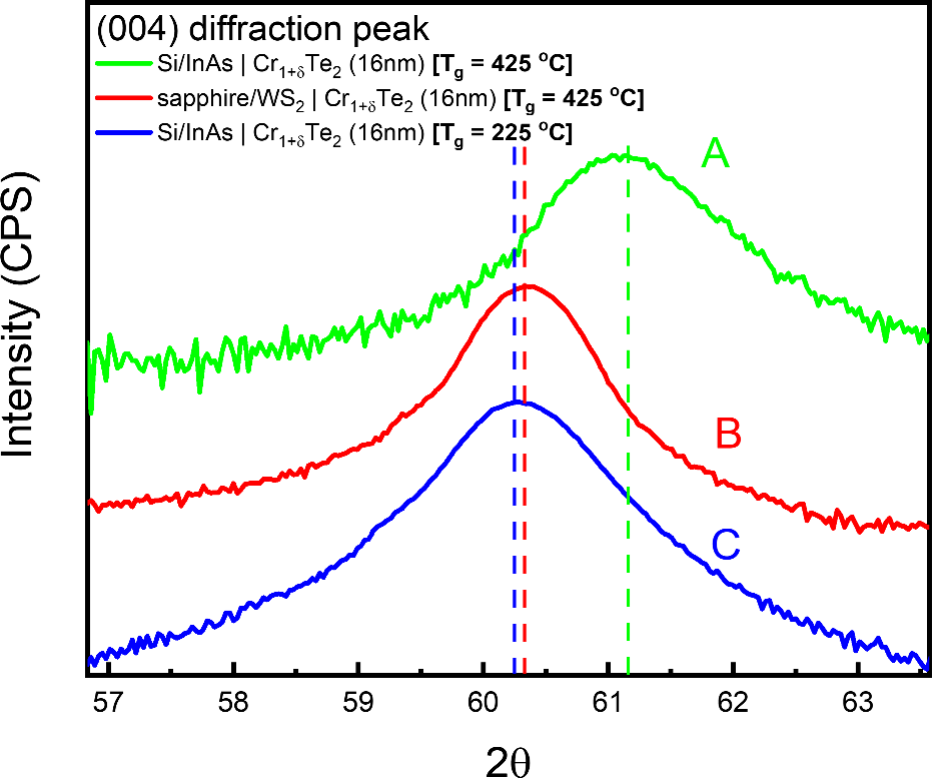
2θ XRD diffractogram of Cr_1+δ_Te_2_ samples. The (004) Bragg diffraction peak exhibits a shift of Δ­(2θ) = 0.9° when growing at elevated temperature on InAs compared to samples grown on InAs at low *T*_g_ and samples grown on sapphire/WS_2_ at high *T*_g_. The interlayer spacing slightly reduces, which results in the *c*-axis contraction by 0.08 Å.

### Magnetic Properties

We have studied the magnetic properties of Cr_1+δ_Te_2_ films with the highest Cr-intercalated content (δ = 0.76) grown on InAs at high *T*_g_ by a combination of SQUID magnetometry and MOKE magnetometry for a thickness series of Cr_1+δ_Te_2_ layers ranging from 2 to 16 nm (see the Supporting Information). In Figure 5, we present the results obtained for the thickest film. The magnetic hysteresis loops acquired by SQUID magnetometry on the 16-nm-thick Cr_1+δ_Te_2_ film (Figure 5a) show that coercivity persists for *T* = 250 K, with a finite moment at zero external magnetic field. The steplike features observed in the magnetization loops at low temperatures (50 and 100 K) may indicate the pinning of magnetic domains. Magnetic switching occurs at different fields, probably due to different coercivities. This behavior has already been observed in Cr_1+δ_Te_2_ and could originate for example from the grain boundaries of rotated domains^49^ like those exhibited in Figure 3c, where magnetic domain pinning could be possible. Similar features have been observed in other literature studies of epitaxial chromium telluride^25,29,50^ and have been attributed to various effects. Among them, pinning and rotated domains and boundaries could be consistent with our experimental findings.

**Figure 5:**
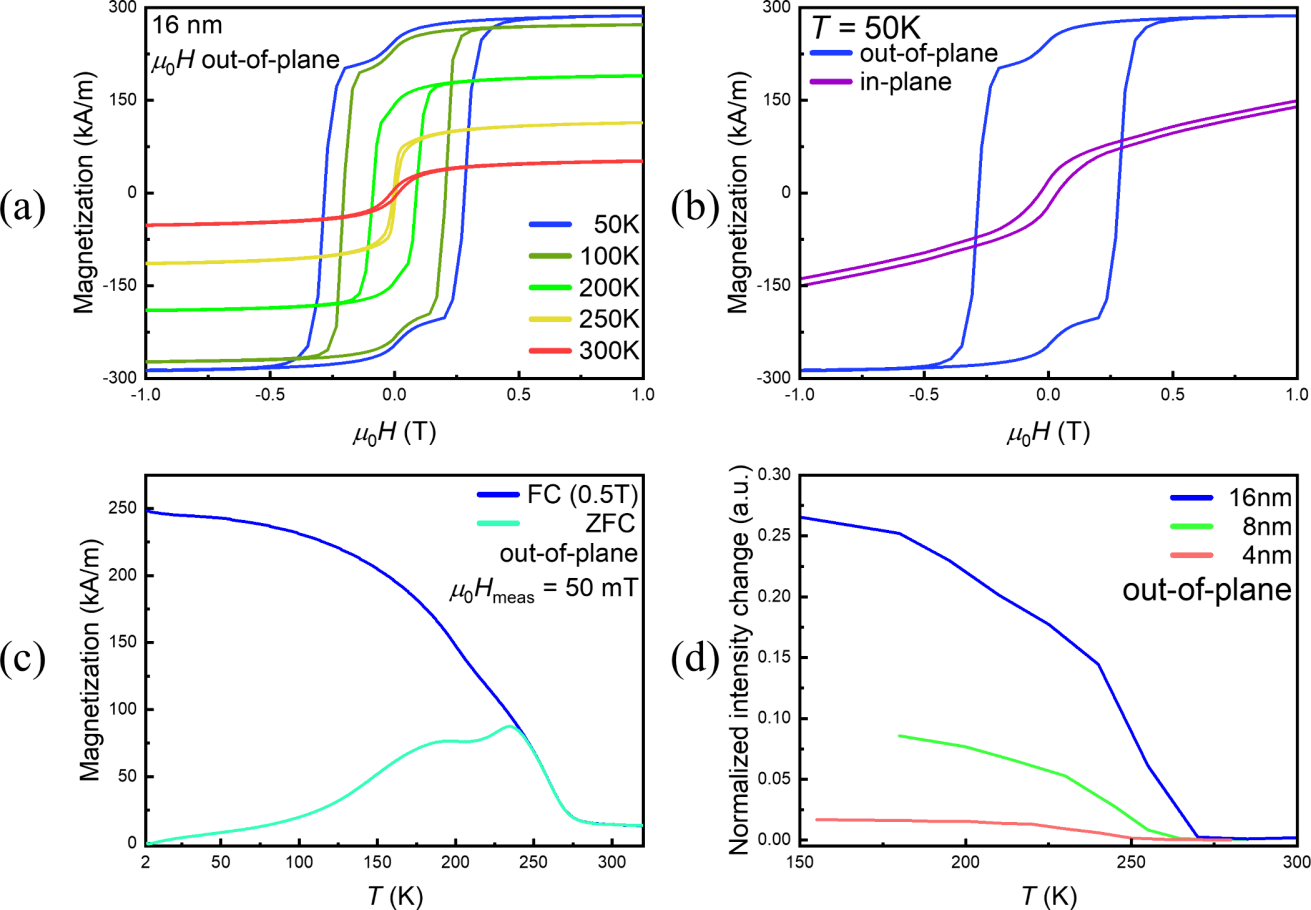
Magnetic characterization of high-*T*_g_ Cr_1+δ_Te_2_ sample on Si/InAs substrate by SQUID and MOKE magnetometry. (a) SQUID magnetic hysteresis loops M­(H) from 50 K to RT with out-of-plane magnetic field for the 16-nm-thick sample. (b) SQUID magnetic hysteresis loops *M*(*H*) acquired at 50 K with an external magnetic field applied in-plane or out-of-plane, reveal that the easy axis of the magnetization lies out of the 16-nm-thick film’s plane. (c) Temperature-dependent out-of-plane magnetization curves *M*(*T*) obtained by SQUID magnetometry for FC (at 0.5 T) and ZFC protocols. Measurements are performed upon warming up with a field of 50 mT (μ_0_H_meas_). (d) Normalized intensity change vs *T* obtained by MOKE indicates a Curie temperature around 260 K for the thickest Cr_1+δ_Te_2_ sample of 16 nm. The intensity change of the reflected light due to Kerr rotation is proportional to the magnetization and it is presented here as a function of temperature.

Magnetic hysteresis loops at 50 K, with an external field applied parallel or perpendicular to the film plane (Figure 5b), reveal that the easy axis of the magnetization is out-of-plane. This is unlike the in-plane magnetic easy axis observed in all other films grown on substrates other than InAs at high *T*_g_, with similarly high Cr intercalation content (see Figure 6). From the in-plane and out-of-plane hysteresis loops in Figure 5d, we can estimate the effective uniaxial magnetic anisotropy energy density *K*_eff_,^51^ at 50 K (further details for the temperature dependence of *K*_eff_ can be found in the Supporting Information), and obtain a value for *K*_eff_ = +0.24 MJ/m^3^. The magnitude of *K*_eff_ compares favorably with the highest *K*_eff_ ≈ +0.6 MJ/m^3^ (or 6 × 10^6^ erg/cm^3^) observed for a Cr_2_Te_3_ film, albeit with characteristically low *T*_C_ ≈ 160–170 K.^27,52^ From the Curie–Weiss law fitting of the temperature-dependent field-cooled (FC) out-of-plane magnetization curve presented in Figure 5c, we determine the *T*_C_ of our 16-nm-thick Cr_1+δ_Te_2_ film to be 258 K (see the Supporting Information (Figure S8) for further details). The deviation at high temperature between FC and zero-field cooled (ZFC) *M*(*T*) curves observed in Figure 5c is indicative of the onset of the strong uniaxial anisotropy in the Cr_1+δ_Te_2_ films under study, while the diminishing magnetization of the ZFC curve at lower *T*, suggests the coexistence of antiferromagnetic (AFM) and ferromagnetic coupling in the Cr_1+δ_Te_2_ film.^18,36^ This overall behavior is remarkably reminiscent of that observed in epitaxial FM thin films with strong PMA which undergo a so-called spin reorientation at intermediate temperature, from a collinear FM toward a noncollinear spin-canted magnetic order.^53^ Thinner layers, down to 4 nm, and measured by MOKE (Figure 5d) show that PMA and a high *T*_C_ of ∼255–265 K are maintained albeit with a reduction of the saturation magnetization as the thickness is reduced. *M*(*H*) hysteresis curves also shown in Figure S6 confirm the robustness of PMA down to ultrathin Cr_1+δ_Te_2_ films of 2 nm. The 16 nm sample grown on InAs has a coercive field of 270 mT at 50 K and the well-shaped hysteretic behavior remains up to 255 K (as shown in Figure S4­(a)). Our results suggest that, by using a suitable InAs substrate, we can achieve strong PMA with high *K*_eff_ and with enhanced *T*_C_ reaching up to 260 K in our Cr_1+δ_Te_2_ epitaxial film.

**Figure 6:**
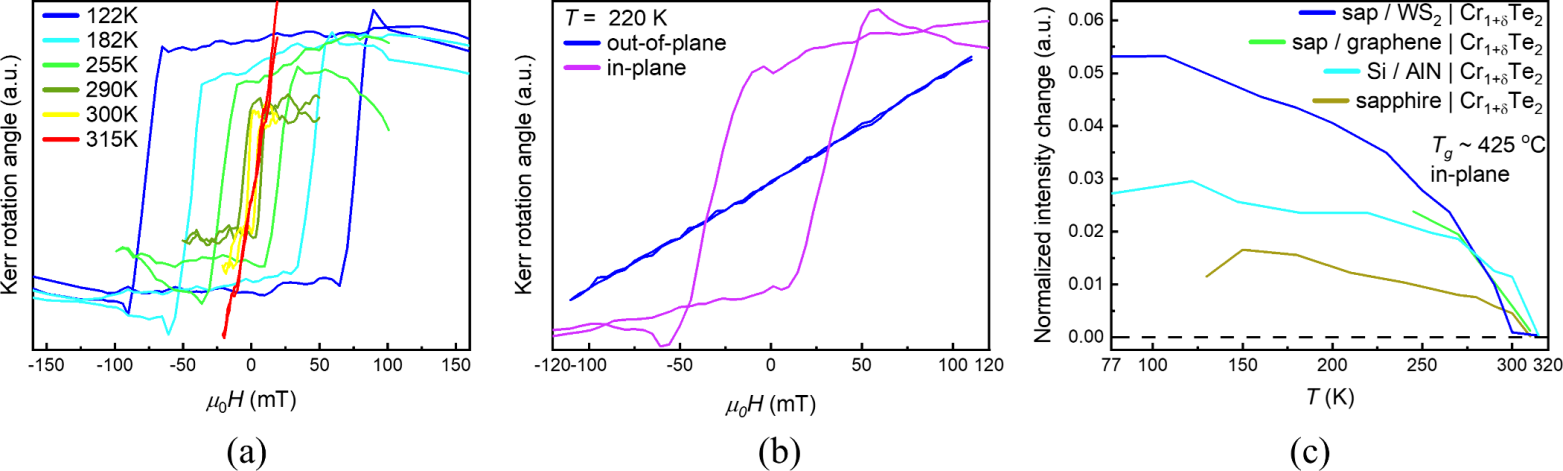
Magnetic hysteresis *M*(*H*), with external magnetic field parallel to the film plane, of high-*T*_g_ Cr_1+δ_Te_2_ samples on (a, b) Si/AlN substrate. Magnetic order is sustained above RT, and Kerr rotation measurements reveal that the easy magnetization axis lies in the plane by comparing signals for in-plane and out-of-plane fields at a given temperature. (c) Normalized intensity change as a function of temperature for high-*T*_g_ Cr_1+δ_Te_2_ on various substrates, except Si/InAs.

MOKE magnetometry data, presented in Figure 6, shows the magnetic properties of Cr_1+δ_Te_2_ layers with thicknesses of 8 and 16 nm, grown on different substrates, at high *T*_g_ and with high intercalated Cr content. Figure 6a shows that the coercivity of a layer grown on Si/AlN substrate persists at *T* > 300 K. Figure 6b shows that a hysteresis loop is obtained only with the external magnetic field parallel to the film plane, indicating that the easy axis is in-plane. To further investigate the influence of the substrate on the magnetic properties, chromium telluride samples grown at a high *T*_g_ of 425 °C on various substrates were studied with MOKE magnetometry as shown in Figure 6c. All samples exhibit in-plane magnetization with *T*_C_ above 300 K. According to the literature^27^ this is expected for Cr_1+δ_Te_2_ compounds with high content of Cr intercalation (δ ≥ 0.44), consistent with the composition measured on our films by RBS (δ ≥ 0.76) for our samples. The results of Figure 6c are in distinct contrast with the high-*T*_g_ grown layers on InAs which have similarly high Cr content but slightly lower *T*_C_ and strong PMA. All investigated substrates except InAs, follow the trend proposed in the literature.^27^ Cr_1+δ_Te_2_ films with lower δ values consistently exhibit PMA with T_C_ below RT, while higher δ values correlate with in-plane magnetic anisotropy and *T*_C_ above 300 K. The results underline that Cr concentration is not the only factor that affects the magnetic properties of the material and reveals the importance of InAs substrate and associated In diffusion to control the magnetic properties of Cr_1+δ_Te_2_ compounds.

By comparing samples grown on InAs substrate at different *T*_g_, we clearly identify that the *T*_C_ of the low-*T*_g_ samples is significantly lower than that of high-*T*_g_ samples, while both samples maintain their PMA character (Figure S10). Thus, revealing that indium plays a significant role in stabilizing the PMA of high-δ Cr_1+δ_Te_2_ films while concomitantly maintaining a relatively elevated value for *T*_C_.

### First-Principles Calculations

To better understand the magnetic properties of high-*T*_g_ Cr_1+δ_Te_2_ on InAs and the beneficial role of *c*-axis contraction on the magnetic anisotropy first-principles calculations were performed. Since the exact crystal structure of our Cr_1+δ_Te_2_ sample is not known and inhomogeneities might occur, we modeled our material by assuming an average structure of CrTe, which is close to the experimental stoichiometry δ = 0.76 indicated by RBS data. For the lattice constants, we used the experimental values, for the samples grown on InAs, obtained from RHEED and XRD data, namely, *a* = 3.87 Å and *c* = 6.15 Å. First, we assume the trivial case of a full spin alignment along the *c*-axis. The value of magnetic moment per Cr atom was found in this case to be close to 4 μ_B_, a value much higher than 1.46 μ_B_ that we observe in our magnetometry experiments. This further indicates that we have a deviation from the classical collinear magnetization state.

As it is already reported, chromium tellurides present complex magnetic configurations that involve spin canting structures along different crystallographic axes.^27,54^ Based on that, the possibility of stabilization of a noncollinear spin state was investigated by exploring the energy landscape as a function of different angles between neighboring spins (Figure 7a). To do so, we performed magnetization constrained DFT calculations,^55^ as implemented in the VASP package. We kept the total magnetic moment fixed and we enforced different angles between neighboring spins in an alternating way along adjacent Cr layers as depicted in Figure 7a. As we can see (Figure 7b, black curve) for ψ = 0, which corresponds to a collinear arrangement, there is a local minimum but the global energy minimum determining the magnetic groundstate is a noncollinear arrangement with the angle between the spins close to ψ = 90°. Further, by comparing the collinear arrangement of the spins along the *c*-axis with the noncollinear one, we found that the canted magnetic structure is the most stable, with an energy difference close to 800 meV, for any given value of angle ψ.

**Figure 7:**
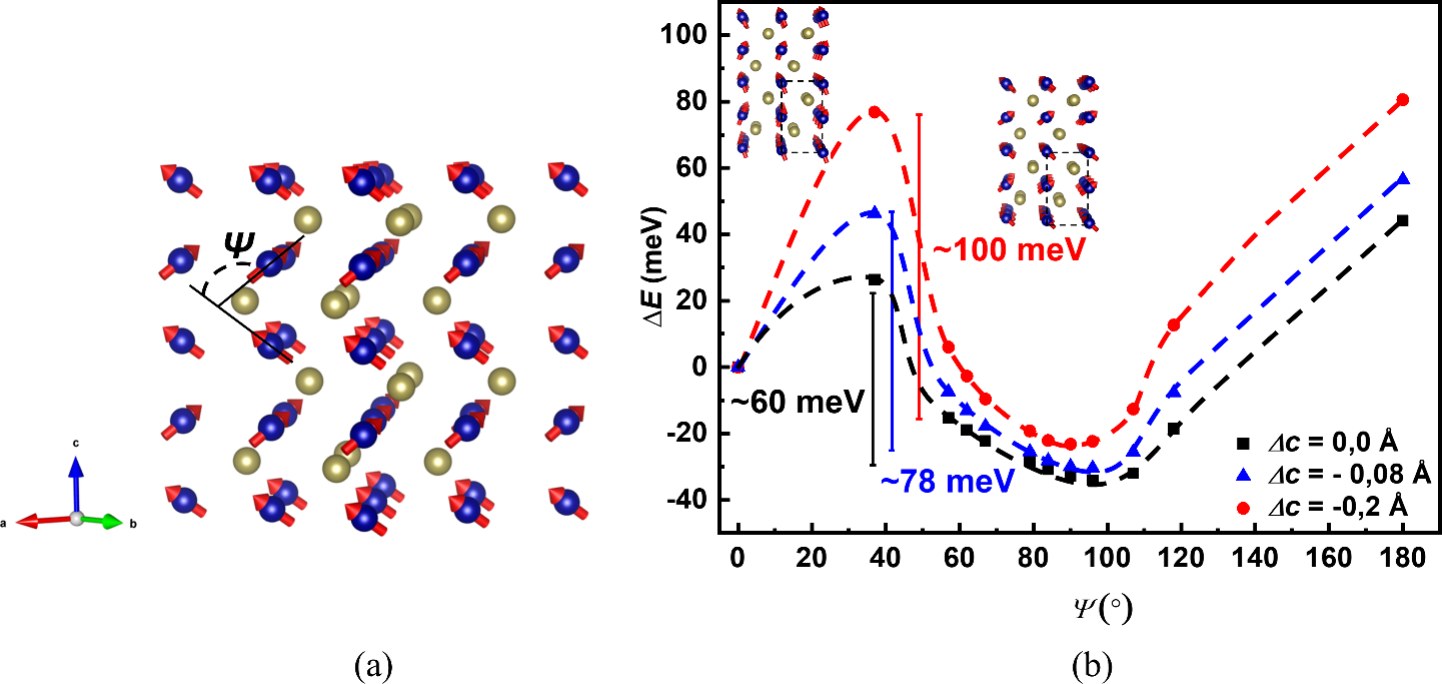
(a) Noncollinear magnetic state. The angle ψ represents the angle between the spins (red arrows) on Cr atoms (blue spheres) in adjacent layers along the *c*-axis. Te atoms are depicted as gold spheres. (b) Variation of system’s total energy as a function of the angle ψ between the adjacent layers’ spins. The collinear configuration where ψ = 0 is taken as a reference energy value. Dashed lines are guides to the eye.

In order to determine the effect of *c*-axis contraction on the stability of this noncollinear phase, we decreased the *c*-axis value by Δ*c* = −0.08 Å and −0.2 Å. As we observe in Figure 7b, the energy barrier between the noncollinear groundstate and the collinear one increases as the *c*-axis contracts. The magnetic groundstate is again close to ψ = 90° for all Δ*c*, although with a noticeable difference in the shape of the curve close to the absolute minimum. There is a small shift of the minimum toward smaller ψ values as *c* is reduced while the minimum becomes better defined and narrower for the maximum studied reduction of Δ*c* = −0.2 Å. It is anticipated that as the out-of-plane lattice constant is reduced, the ferromagnetic exchange interaction between the spins in neighboring layers is strengthened and, on average, becomes dominant over the antiferromagnetic exchange interaction of the interlayer nearest neighbor.^27^ This favors an overall ferromagnetic spin arrangement in the out-of-plane direction, which could explain the PMA, at odds with the expectation of an in-plane anisotropy for this Cr-rich Cr_1+δ_Te_2_ compound. The tentative spin-canted order proposed here offers a worthy goal to experimentally verify, via, e.g., neutron diffraction, which remains generally challenging for small volume samples.

## Discussions and Conclusions

In this work, we report on a new Cr-rich phase of the vdW layered Cr_1+δ_Te_2_ (δ ≥ 0.76) compounds, nearing the stoichiometric composition Cr_7_Te_8_. The new epitaxial phase produced by MBE at high growth temperature has a strong PMA with a relatively high *T*_C_ reaching up to 260 K, in contrast to other Cr-rich phases with similar composition and thickness which present in-plane anisotropy with (near) room temperature *T*_C_. The new phase presents a distinct Moiré pattern and a contraction of its *c*-axis, which are attributed to indium diffusion from the InAs substrate during the high *T*_g_ growth. In brief, indium acts as a surfactant inducing a coherently, strained Cr_1+δ_Te_2_ layer with reduced out-of-plane lattice parameter. This offers a new way to modify the magnetic anisotropy and the *T*_C_ in addition to the typically employed control of magnetic properties by means of Cr composition variations.^27,46^ To gain deeper insight, we discuss below the results considering the saturation magnetization and possible geometrical, shape and surface contributions, as well as the interplay between the AFM and FM interactions in the CrTe compound.

The structural parameters and the magnetic properties of several Cr_1+δ_Te_2_ films discussed in the sections above are summarized in Table 1. The magnetic moment per Cr atom *m*_Cr_ extracted from saturation magnetization measurements *M*_s_ are compared with theoretical values. Values of *m*_Cr_ for the stoichiometries Cr_7_Te_8_, Cr_3_Te_4_ ^24,56^ and Cr_2_Te_3_ ^52,57^ phases taken from the literature are summarized in the last column of Table 1. We note that the value for Cr_7_Te_8_ is not available in the literature, such that we chose to present *m*_Cr_ from CrTe,^15^ the closest stoichiometric compound with the most intercalated chromium. It is worth noting that the Cr-rich sample S1 grown at high *T*_g_ on InAs substrates has the lowest *m*_Cr_ value, despite the fact that it is the denser layer, with respect to the Cr atoms. In addition, the *m*_Cr_ value of 1.46 μ_Β_ is significantly lower than the theoretical value of 3.95 μ_Β_.^15^ This discrepancy is attributed to the noncollinear arrangement of magnetic moments at neighboring layers as reported previously,^27,54^ especially for the Cr_2_Te_3_ compound.^20,58^ The canting of the magnetic moments is considered to be a result of the competition between antiferromagnetic and ferromagnetic interlayer coupling. It has been reported that the AFM exchange interaction dominates the nearest interlayer neighbors while the FM one prevails over the intralayer and interlayer Cr–Cr interactions at longer distances. This could explain the in-plane magnetization observed in CrTe and more generally in the high-δ Cr_1+δ_Te_2_ compounds.^27,54^ As the *c*-axis—and, hence, the interlayer Cr–Cr atom distance—is reduced, the exchange interactions could change in favor of an enhanced FM coupling, resulting in the observed PMA, albeit with reduced saturation magnetization.

**Table 1:** Samples of Cr_1+δ_Te_2_ Studied in the Present Work

	substrate	*T*_g_ (°C)	δ	nearest stoichiometric compound (Cr_1+δ_Te_2_)	nominal or RBS thickness (nm)	*M*_s_/*V* (kA/m) SQUID (@50 K)	Cr magnetic moment, *m*_Cr_ (μ_b_)	*m*_Cr_ literature values (μ_b_)
S1	Si(111)/InAs(111)	425	0.76 (RBS)	Cr_7_Te_8_	14.7 (RBS)	289.1	1.46	3.95
MBE 2892								
S2	Si(111)/InAs(111)	425	0.76	Cr_7_Te_8_	16 (nominal)	–	–	3.95
MBE2886								
S3	Si(111)/InAs(111)	425	0.76	Cr_7_Te_8_	8 (nominal)	253.6	1.28	3.95
MBE 2888								
S4	Si(111)/InAs(111)	425	0.76	Cr_7_Te_8_	4 (nominal)	399.0	2.02	3.95
ΜΒΕ 2896								
S5	Si(111)/InAs(111)	425	0.76	Cr_7_Te_8_	2.5 (nominal)	383.6	1.94	3.95
MBE 2898								
S6	Si(111)/AlN	425	0.44 (RBS)	Cr_3_Te_4_	17.6 (RBS)	363.1	2.32	3.32
MBE 2911								
S7	Si(111)/InAs(111)	225	0.36 (RBS)	Cr_2_Te_3_	20.4 (RBS)	258.0	2.39	2.65
MBE 2889								
S8	Si(111)/AlN	225	0.32 (RBS)	Cr_2_Te_3_	19.7 (RBS)	–	–	2.65
MBE 2912								

One may seek alternative explanations for the strong PMA in our films taking into account shape and geometrical effects associated with demagnetization. As generally accepted, in thin films, an in-plane easy axis (or easy plane) is favored to avoid a large demagnetization energy in the out-of-plane direction.^59^ We argue that, in our films, the much-reduced saturation magnetization due to the spin canting results in weaker demagnetization fields such that the PMA character is preserved. It should be noted however that significant demagnetization in chromium telluride films is not expected to play a major role due to weak magnetic dipolar interactions.^27^

Other explanations for the strong PMA observed may be related to the presence of large out-of-plane surface anisotropic contributions *K*_s_ to the total *K*_eff_. A large *K*_s_ may come from the top surface layer responsible for the observed Moiré pattern (Figure 3). The value of *K*_s_ may outweigh the possible negative contributions *K*_υ_ from magnetocrystalline anisotropy to give an overall positive *K*_eff_ value. However, this would be expected to occur mostly in the ultrathin film limit where surface effects dominate and is thus less significant in our relatively thick films of 16 nm.

In summary, the unexpectedly strong PMA in the Cr-rich Cr_1+δ_Te_2_ compounds reported here, with a relatively high *T*_C_, is attributed to the interplay between nearest neighbor interlayer AFM and longer-range FM interactions. The latter are thought to be enhanced by the *c*-axis contraction as a result of indium diffusion from the InAs substrate. First-principles calculations further hint at a potential staggered spin-canted order along the *c*-axis. Our work paves the way toward a synthesis route able to control the composition and magnetic properties of Cr_1+δ_Te_2_ 2D vdW ferromagnets through appropriate choices of the substrate, the growth temperature and potentially the use of an indium flux during growth.

## Supplementary Material



## Notes

Additional experimental details, data concerning structural and physical properties of the materials mentioned in the manuscript (PDF)

## ∇

For Panagiotis Pappas: CEA, IRIG, SPINTEC, 38000 Grenoble, France. Email: panagiotis.pappas@cea.fr

## △

For Elli Georgopoulou-Kotsaki: Univ. Grenoble Alpes, CEA, CNRS, Grenoble INP, SPINTEC, 38000 Grenoble, France. Email: elli.georgopouloukotsaki@cea.fr.
