# Supplementary material for: Substrate-Driven Stabilization of Perpendicular Magnetic Anisotropy and Near-Room-Temperature Ferromagnetism in Cr-Rich Cr1+δTe2 Films
Source: J Phys Chem C Nanomater Interfaces. 2025 Sep 5;129(37):16709–18. doi: 10.1021/acs.jpcc.5c02927 (PMC12451664; doi:10.1021/acs.jpcc.5c02927)
Supplement: Supplementary file 1 [file jp5c02927_si_001.pdf]

Supplementary Information

Substrate-Driven Stabilization of Perpendicular Magnetic Anisotropy and Near-Room-Temperature Ferromagnetism in Cr-Rich $Cr_{1+\delta}Te_2$ Films

Akylas Lintzeris [1,2,], Polychronis Tsipas[1], Shanshan Guo[3], Panagiotis Pappas[1], Elli Georgopoulou–Kotsaki[1], Ilya Kostanovski[4], Claudia Felser[3], Edouard Lesne[3], Hanako Okuno[5], Athanasios Dimoulas[1]*

[1]Institute of Nanoscience and Nanotechnology, National Center for Scientific Research "Demokritos", 15310 Athens, Greece

[2]School of Applied Mathematical and Physical Sciences, National Technical University of Athens, 157 80 Athens, Greece

[3]Max Planck Institute for Chemical Physics of Solids, 01187 Dresden, Germany

[4]Max Planck Institute of Microstructure Physics, 06120 Halle (Saale), Germany

[5]CEA, IRIG-MEM, Université Grenoble Alpes, 38000 Grenoble, France

Corresponding Author

*Akylas Lintzeris a.lintzeris@inn.demokritos.gr

XPS

We have performed in-situ XPS using a Mg source to analyze the elemental composition of the sample. Additionally, we present the Auger peak for chromium as the Cr $3d_{3/2}$ peak region overlaps with the Te $3d_{5/2}$. XPS Auger Peak of chromium remains unchanged regardless of the thickness of the samples grown on Si(111) / InAs substrate.

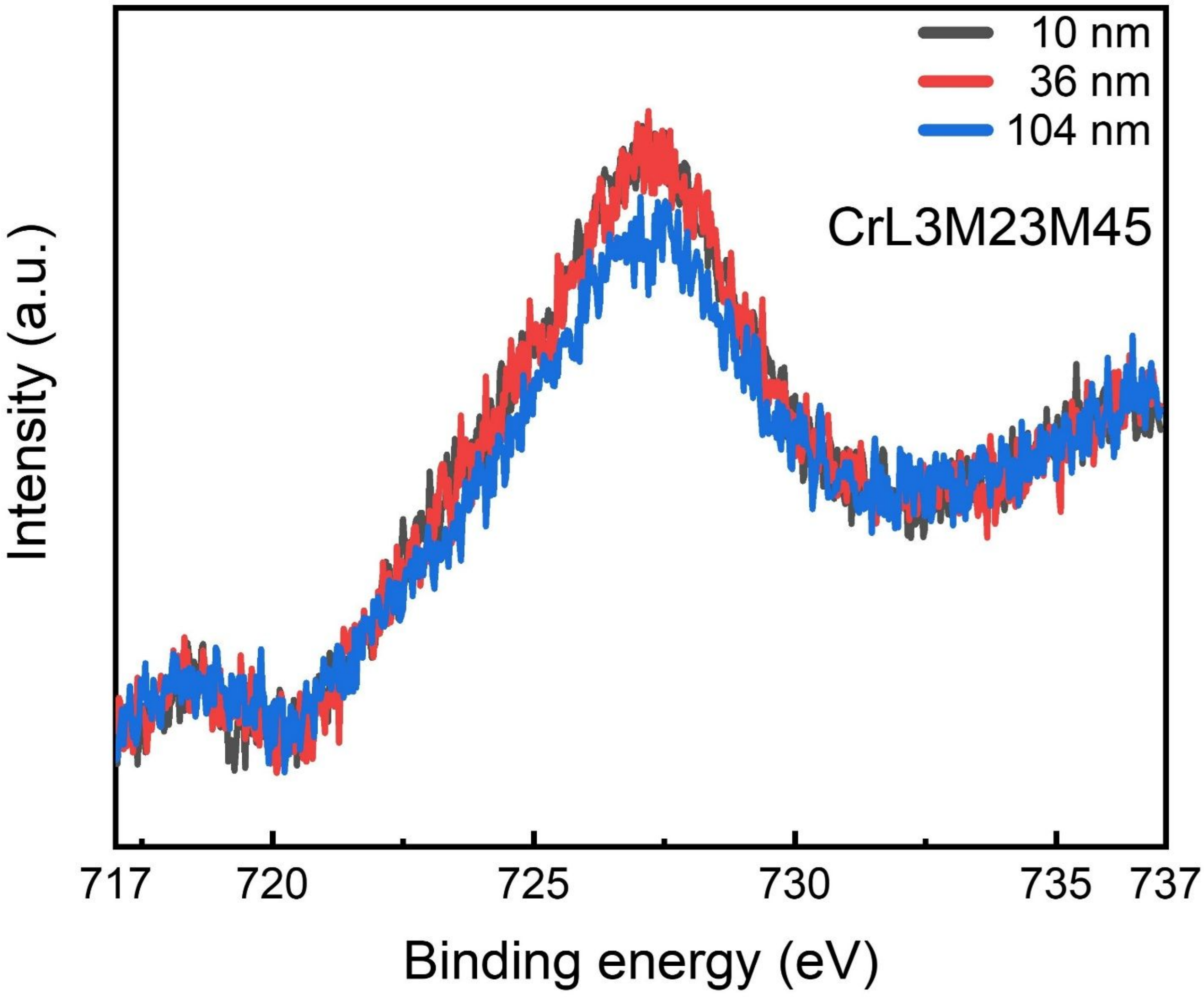

Figure S1. X-ray photoemission spectroscopy (XPS) spectrum of Cr Auger peak that do not change significantly with the thickness of the sample.

RHEED

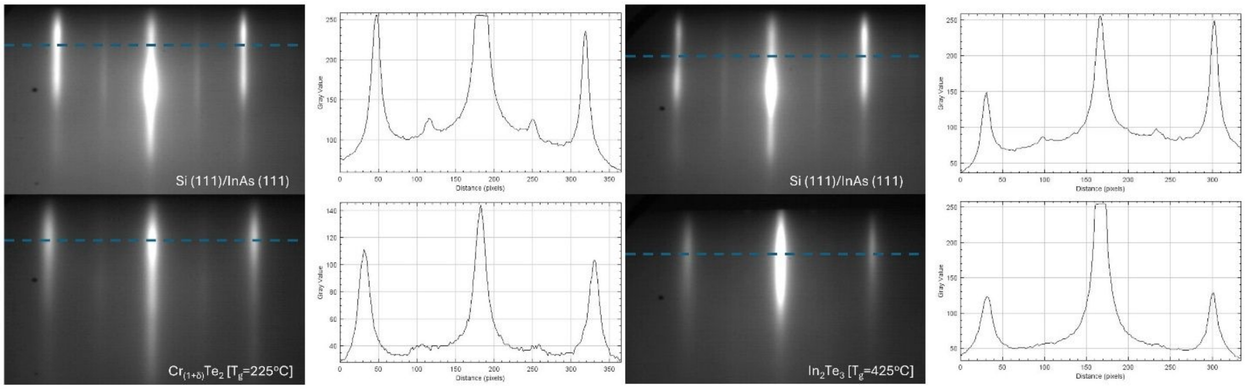

(a) (b)

Figure S2. RHEED patterns before (top) and after (bottom) deposition of 16nm $Cr_{1+\delta}Te_2$ samples.

The line-scan of each RHEED pattern is presented alongside. Lattice constant *a* of InAs is 4.284 Å and is used to calculate the corresponding lattice constants of the top layer after deposition. **(a)** The sample grown on 225°C exhibits a *2x2* reconstruction which is compatible with that of $Cr_{1+\delta}Te_2$. The lattice constant *a*, is estimated 3.87 Å. **(b)** High T_g grown sample does not exhibit any surface reconstructions, and the lattice constant *a* is estimated 4.364 Å.

InAs surface of very good quality is observed on the all the Si(111) / InAs substrate. *2x2* reconstruction at the surface of InAs indicates the absence of oxidation and impurities that could degrade the quality of the material that will be grown epitaxially on these substrates.

Different phases of $Cr_{1+\delta}Te_2$ give rise to different surface reconstructions that are characteristic for each separate phase. Low T_g samples manifest *2x2* (Cr_5Te_8) and *1x1* ($CrTe_2$) and on the contrary, high T_g chromium tellurides exhibit *2x1* (Cr_3Te_4) or *(√3x√3)* R30° (Cr_2Te_3) reconstructions[1]. As shown in Sup. Fig. 2 (a), low T_g sample on InAs, the dominant *1x1* pattern attributed to $CrTe_2$ is clearly observed and only a faint *2x2* superstructure is present. Combining those two observations, one can assume that there is low self-intercalation of Cr atoms resulting in relatively small δ factor with values $\delta \leq 0.3$. The $Cr_{1+\delta}Te_2$ phases which are consistent with this value are Cr_2Te_3, Cr_5Te_8 and $CrTe_2$. High T_g samples do not exhibit any surface reconstruction, and a significantly larger lattice constant *a* is observed.

XRD

X-ray diffraction patterns of samples grown on InAs substrate are presented in Sup. Fig. 3. Different $Cr_{1+\delta}Te_2$ stoichiometric phases have very similar crystal structures and lattice parameters[2] so it is rather difficult to distinguish the exact stoichiometry only from an XRD pattern. In this case, one can use the XRD patterns to confirm the presence of the trigonal phase of $Cr_{1+\delta}Te_2$ in all samples regardless of the growth temperature. The trigonal phase Cr_3Te_4 compound can be excluded due to the lack of the predicted diffraction peaks in the pattern (Sup.Fig.3). In the spectrum of the high T_g sample capped with aluminum, a peak at $2\theta = 32.93^o$ emerges that can be attributed to a crystalline In-Al-Te phase formed after the deposition of the Al cap layer, which is supported from HRTEM cross-section images. This peak is absent in the W

capped sample supporting the assumption that a reaction between In, Te and Al is taking place to form the crystalline phase. Samples capped with Al also exhibit a diffraction peak in $2\theta = 38.22^o$ which is attributed to (1 1 1) diffraction of crystalized aluminum.

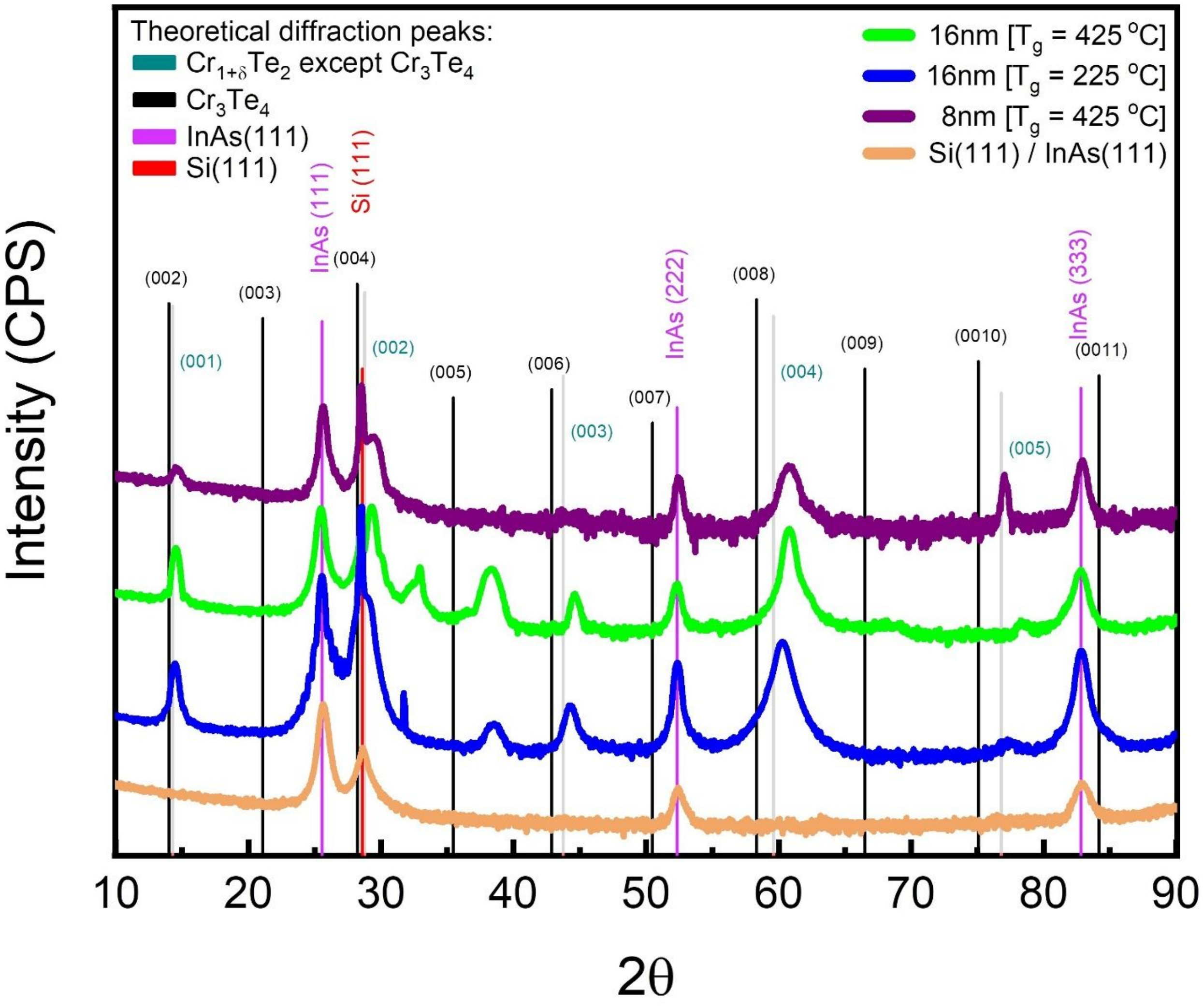

Figure S3. X-ray diffraction (XRD) pattern of samples grown on InAs substrate at both low and high T_g. Blue pattern corresponds to a Si(111) / InAs(111) substrate measured before synthesis where the diffraction peaks of Si(111) and InAs(111) are present.

RBS

Rutherford Backscattering Spectroscopy (RBS) was utilized to get information about the stoichiometry of the grown films. Data was measured using commercial ion beam accelerator system made by NEC set for He+ beam with 1.9 MV energy with 20 nA sample current. Spectra measured at 169 degrees scattering angle, over an area of approx. 2 mm and with the total dose of 40 μQ were processed in SimNRA software. The resulting compositions are presented in the Sup. Table 1. The data acquired from low and high T_g samples on Si (111) /InAs and Si (111) / AlN indicate that the main trend concerning the growth temperature and stoichiometry for epitaxial chromium tellurides is confirmed in our samples.

Substrate	Structure	T_g [^{o}C]	δ factor
Si (111) / InAs	$Cr_{1+\delta}Te_2$ (≃16nm) I Al	225	0.36
Si (111) / InAs	$Cr_{1+\delta}Te_2$ (≃16nm) I Al	425	0.76
Si (111) / AlN	$Cr_{1+\delta}Te_2$ (≃16nm) I Al	225	0.32
Si (111) / AlN	$Cr_{1+\delta}Te_2$ (≃16nm) I Al	425	0.44

Table S1 Rutherford backscattering spectrometry reveals the exact stoichiometry, δ-factor, of the samples.

Low T_g samples on both substrates have relatively small amount of intercalated chromium atoms compared to the high T_g samples. InAs grown samples exhibit significantly larger number of intercalants. The stoichiometry of the sample is close to the Cr_7Te_8 compound.

Magnetic Properties

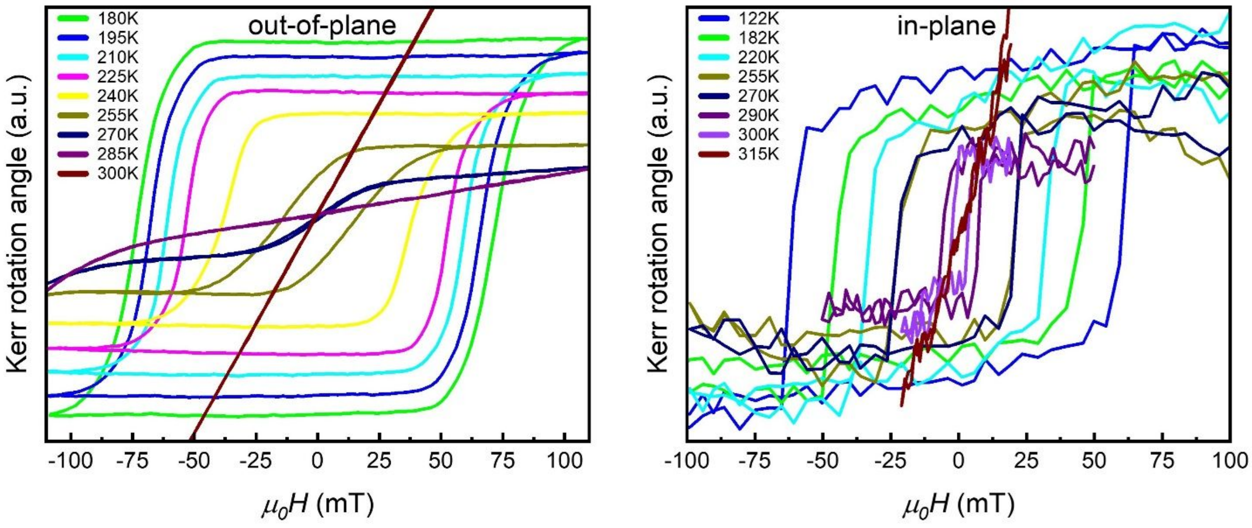

(a) (b)

Figure S4 MOKE magnetometry. The hysteresis loops of the complete temperature range, from 125 K to 315 K. The loops are well shaped with high coercivity at lower temperature. **(a)** Sample grown on InAs substrate has easy magnetization axis out of the film plane at 260 K the coercive field is below 1 mT. down to 4 nm film and below that decreases to 160 K. **(b)** Sample grown on Si/AlN substrate on the same high T_g exhibits in-plane anisotropy with a ferromagnetic hysteresis loop visible at 300 K.

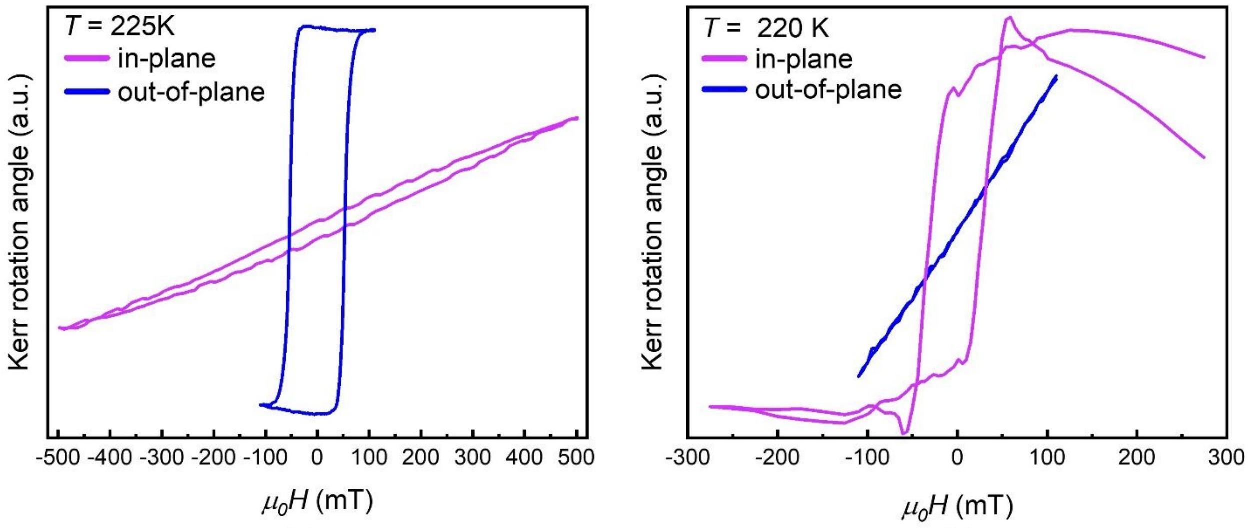

(a) (b)

Figure S5 MOKE magnetometry magnetic hysteresis loops, in polar and longitudinal mode with external field vertical and parallel to the film plane respectively. Both samples are grown at T_g= 425 °C on different substrates. **(a)** Sample grown on Si/InAs exhibits PMA. A rectangular hysteresis loop is acquired with *H*//c and on the contrary, applying *H*//ab, saturation of the sample is not achieved with the available magnitude of magnetic field. **(b)** Conversely, samples grown on Si/AlN exhibit in-plane magnetic anisotropy with a magnetic hysteresis loop when *H*//ab. The sample is not saturated out-of-plane and thus no hysteresis is acquired with the available external magnetic field perpendicular to the film plane.

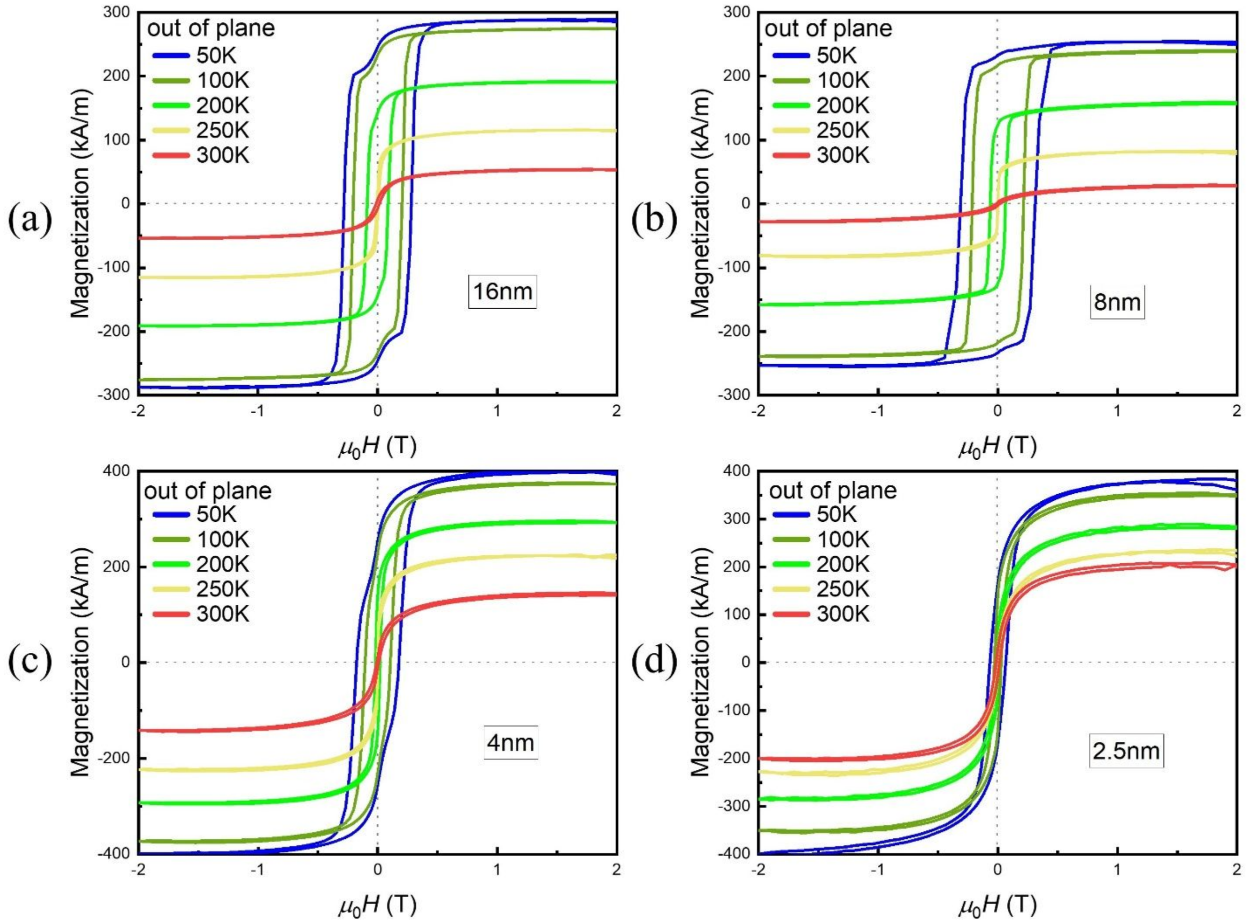

Figure S6 Thickness dependency of magnetic hysteresis loops measured with SQUID for samples of **(a)** 16 nm, **(b)** 8 nm, **(c)** 4 nm, **(d)** 2.5 nm thickness grown on InAs at high T_g.

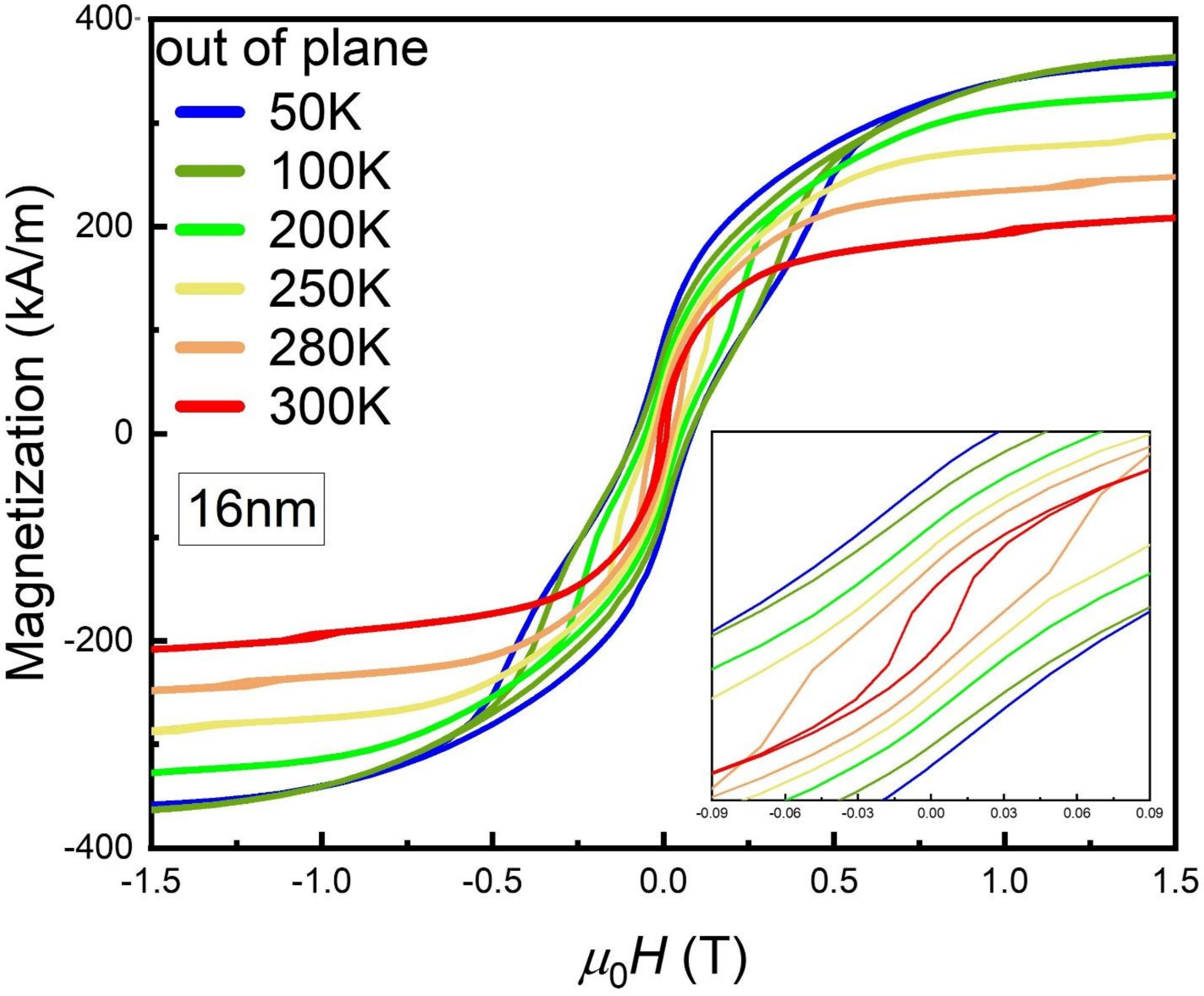

Figure S7. Hysteresis loops for various temperatures of 16 nm sample grown on Si/AlN at high T_g. External magnetic field is applied along the hard axis of the film magnetization, perpendicular to the surface. On the **inset**, a clear hysteresis loop is observed at room temperature (300 K).

Determination of the Curie Temperature (T_C)

To accurately determine the Curie temperature of the samples we follow the Curie-Weiss law to estimate the temperature at which the system undergoes a ferromagnetic transition. The temperature dependence of magnetization in Sup. Fig. 8 is fitted with Curie-Weiss law and T_C is around 258 K.

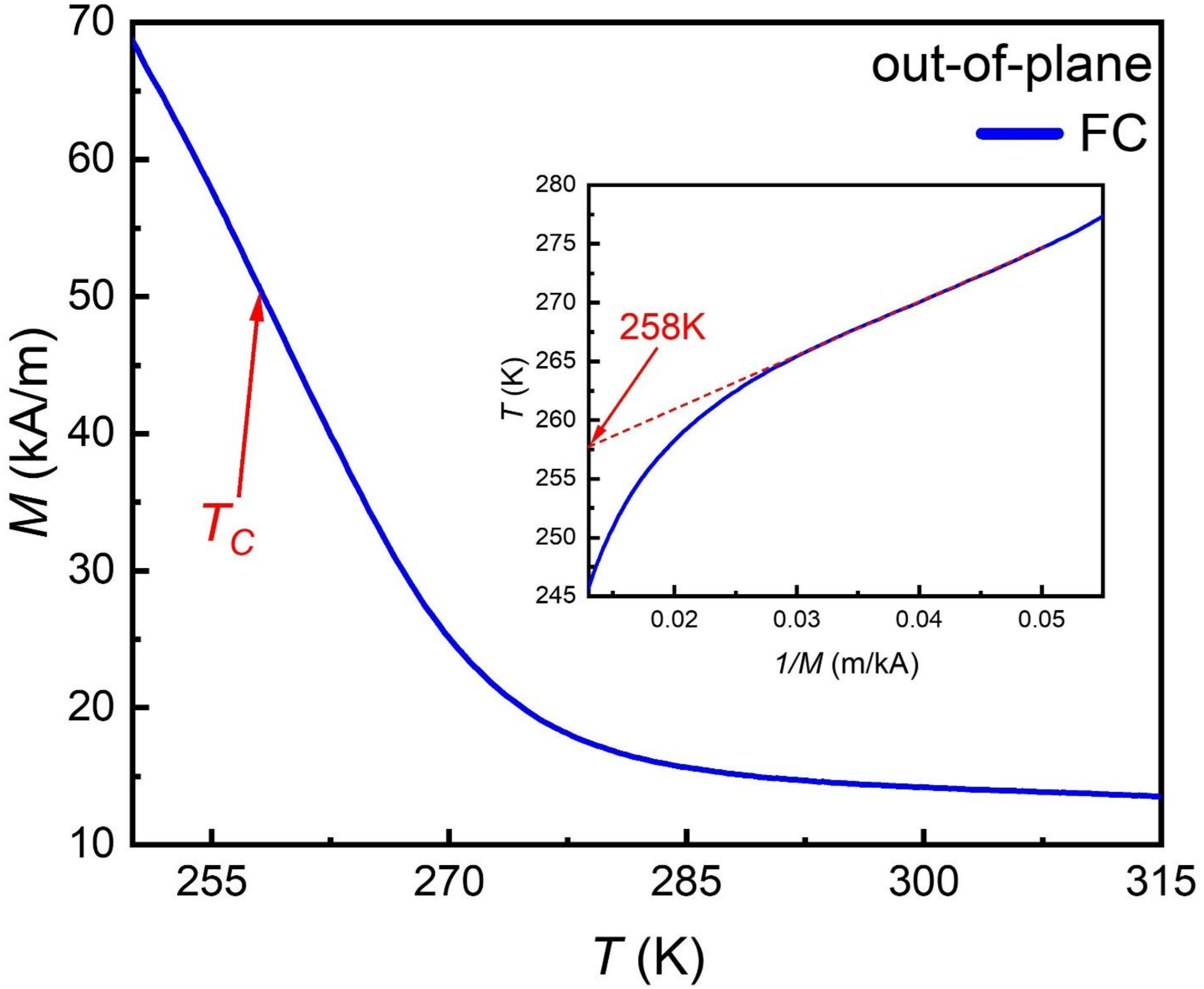

Figure S8. Temperature dependence of magnetization. On the **inset**, the result of the Curie-Weiss law fitting is presented and the T_C is determined to be 258 K.

Magnetic anisotropy calculation

The magnetic anisotropy energy (E_a) can be obtained by comparing the areas under the magnetization vs external magnetic field (M-H) hysteresis loops, measured with parallel and perpendicular to the ferromagnetic layer plane, external magnetic field. The easy axis of the samples' magnetization is perpendicular to the film's plane and the hard axis is parallel. Fig. 5 (b) exhibits the magnetic hysteresis loops for the two different magnetic field configurations at 50 K measured with SQUID.

Anisotropy energy E_a is given from the following equation:

$$E_a = \int_{0[hard-axis]}^{M_s} HdM - \int_{0[easy-axis]}^{M_s} HdM \qquad (1)$$

Where M_s is the saturation magnetization.

The anisotropy constant K_{eff}, can be calculated according to the equation:

$$K_{eff} = \frac{E_a}{V} \qquad (2)$$

Where V is the volume of the ferromagnetic layer estimated from the samples' lateral dimensions and thickness data obtained from the TEM and RBS measurements. We calculate a value K_{eff} $= +2.4 * 10^6 \, {}^{erg}/_{cm^3}$ that confirms the presence of PMA.

Supplementary Figure 9 presents the calculation of the effective uniaxial magnetic anisotropy energy density K_{eff} as a function of temperature, for the sample grown on InAs at high T_g. K_{eff} becomes larger as the temperature decreases, indicating the increase of the anisotropy.

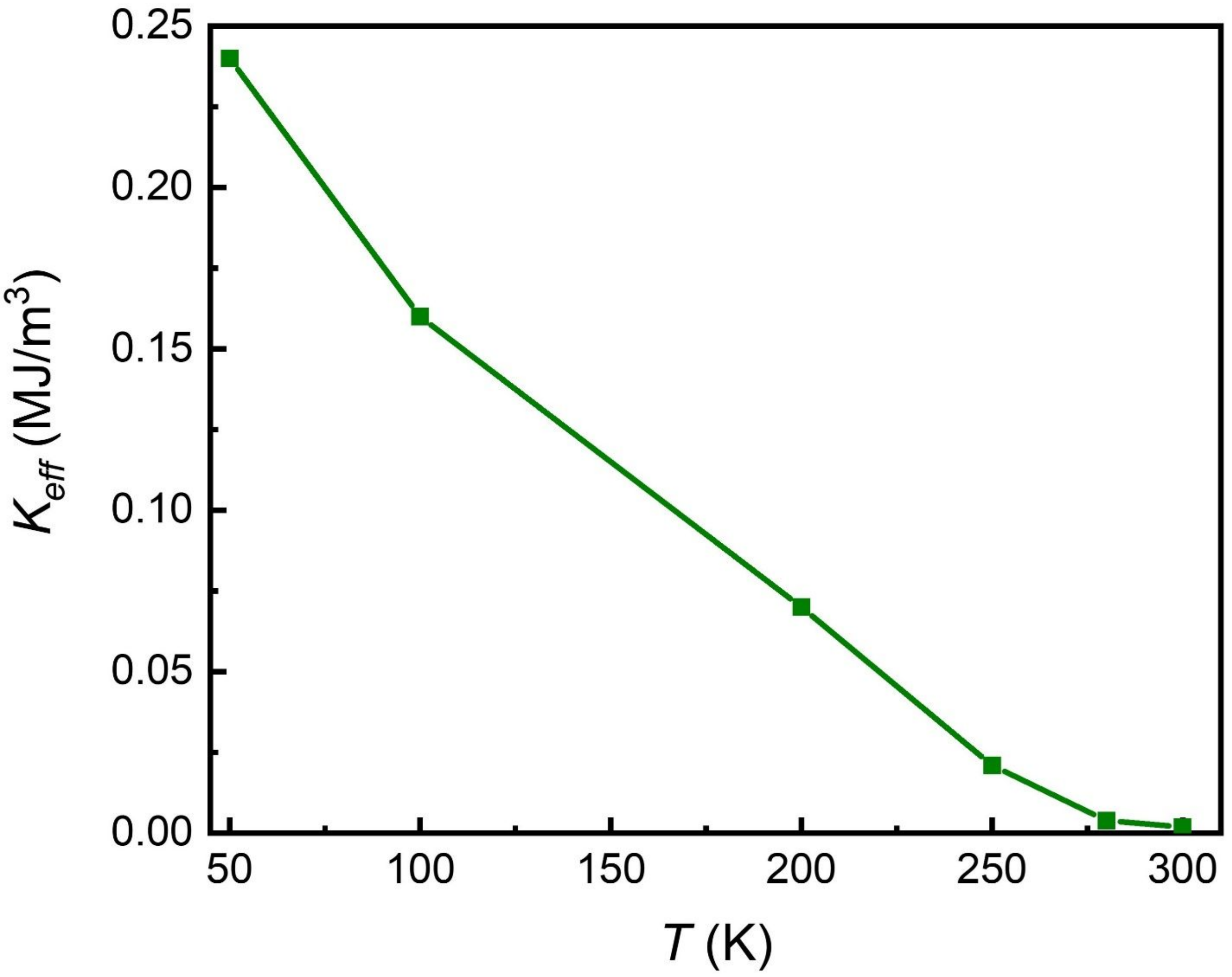

Figure S9. Effective uniaxial magnetic anisotropy energy density K_{eff} as a function of temperature.

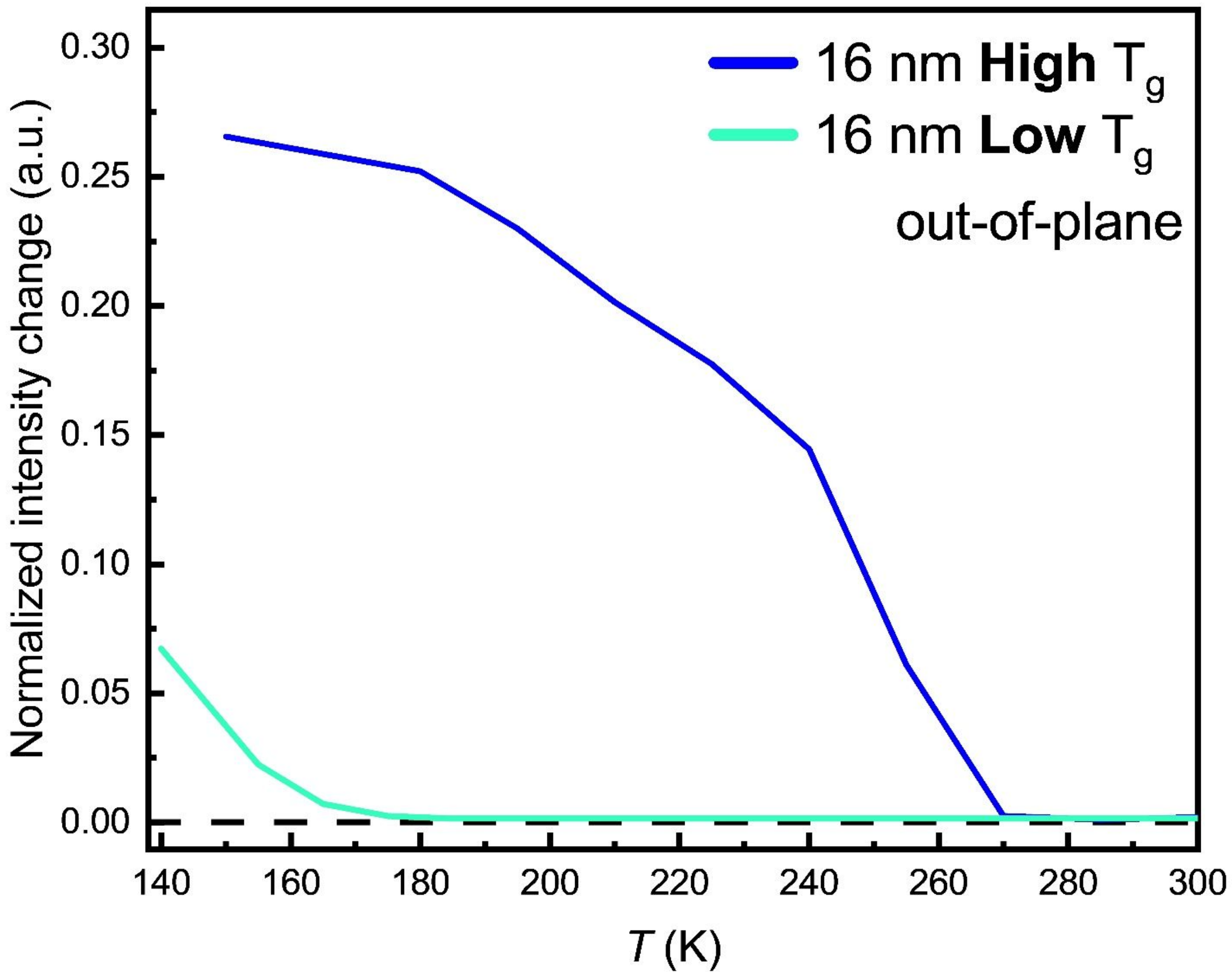

Figure S10. Samples grown on lower temperature have significantly lower T_C than those grown on high T_g and the easy axis of the magnetization is out of the film plane in both cases.

Magnetic moment calculation

To determine the magnetic moment per Cr atom (a_{Cr}) from our SQUID magnetometry measurements, we used the following equation:

$$\frac{M_s}{V_{sample}} = \frac{n_{Cr} \cdot \mu_B \cdot a_{Cr}}{V_{unit\ cell}} \quad (3)$$

$$a_{Cr} = \frac{M_S \cdot V_{unit\ cell}}{n_{Cr} \cdot \mu_B \cdot V_{sample}} \quad (4)$$

Where M_s is the saturation magnetization measured by SQUID magnetometry, V_{sample} is the volume of the ferromagnetic layer, calculated from its thickness and lateral dimensions, n_{Cr} is the number of chromium atoms per unit cell in the nearest stoichiometric compound (CrTe, Cr_3Te_4, Cr_2Te_3, Cr_5Te_8, $CrTe_2$), $V_{unit\ cell}$ is the unit cell volume of the corresponding stoichiometric compound and μ_B is the Bohr magneton.

To evaluate α for each sample, we first determined the δ factor from the available RBS measurements to estimate the actual Cr-to-Te ratio. We then identified the nearest stoichiometric phase to this δ value and used its crystallographic parameters (unit cell volume and number of Cr atoms per unit cell) for the calculation.

This method enables an approximate estimation of the magnetic moment per Cr atom, under the assumption that the magnetic contribution arises primarily from the Cr atoms and that the structural properties of the sample closely resemble those of the selected reference compound. While this introduces some approximation, especially in the presence of Cr intercalation or structural disorder, the calculated values remain meaningful for comparing samples and identifying trends in magnetic behavior across different growth conditions.

Magnetization reversal imaging with MOKE

Magnetization reversal imaged for InAs grown sample at 225K at Sup. Fig. 11. At first, the sample is saturated with 110 mT external perpendicular magnetic field. The saturated state (point A) is a single domain phase which is rendered in dark contrast. Decreasing the external magnetic

field with small steps just before -50 mT the first reversed domains are observed (point B). Continuing the magnetic field steps the film is in a mixed state of "up" and "down" facing magnetic domains slightly lower than -50 mT (point C). In that state, light-contrast domains occupy approximately half of the entire surface. Reaching the opposite saturation state at -110 mT the whole surface exhibits light-contrast and the magnetization reversal has been completed. The domains are better visible at lower temperatures where the magnetization of the sample is stronger but persists until T_C.

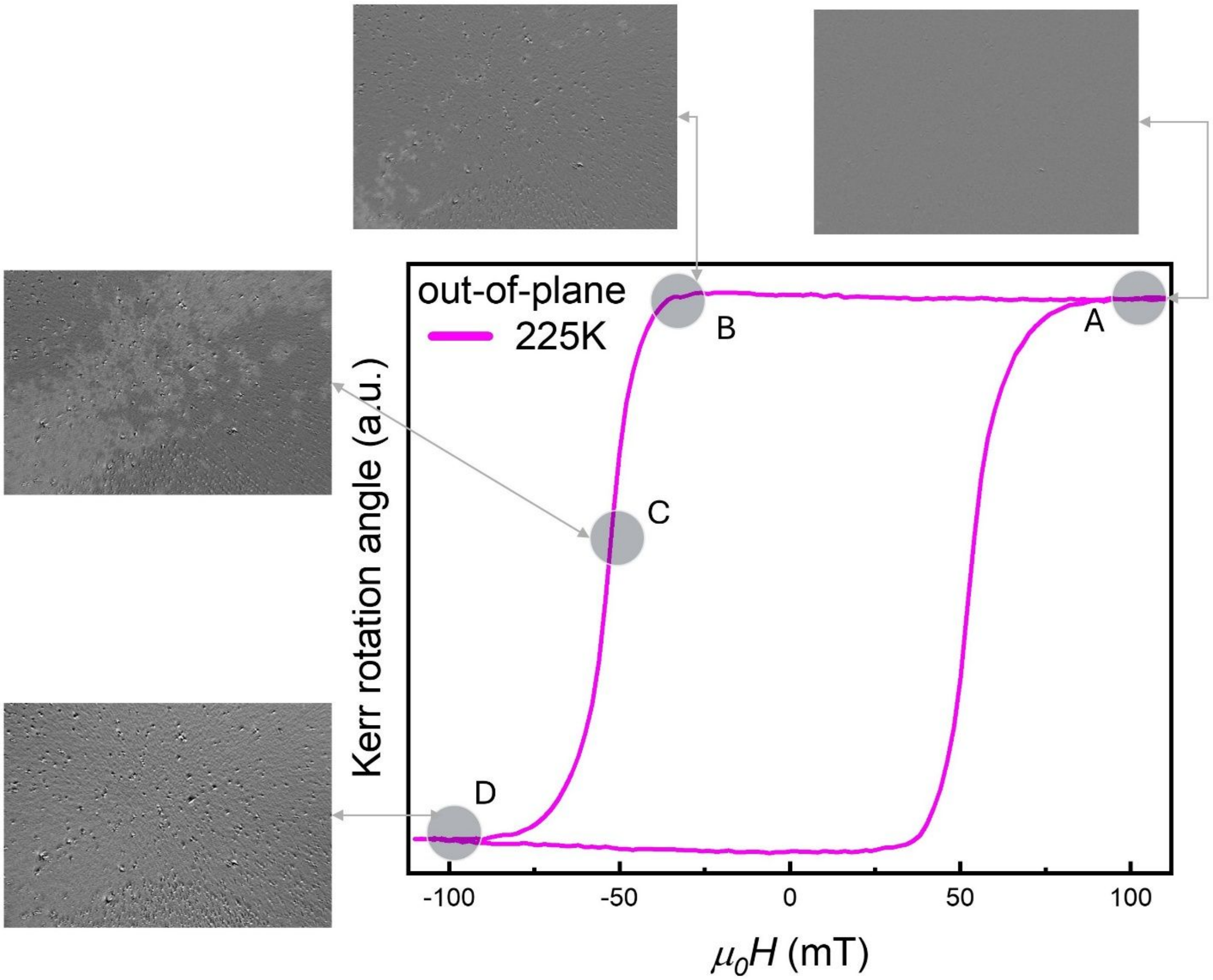

Figure S11. MOKE magnetic domain propagation imaging at points "A", "B", "C", "D" on hysteresis loop for the 16nm $Cr_{1+\delta}Te_2$ taken at 225 K. Images indicate the film's magnetization reversal perpendicular to the plane. The reversal is carried out abruptly and that results in a relatively square hysteresis loop characteristic of a strong PMA material.

REFERENCES

(1) Lasek, K.; Coelho, P. M.; Zberecki, K.; Xin, Y.; Kolekar, S. K.; Li, J.; Batzill, M. Molecular Beam Epitaxy of Transition Metal (Ti-, V-, and Cr-) Tellurides: From Monolayer Ditellurides to Multilayer Self-Intercalation Compounds. *ACS Nano* **2020**, *14* (7), 8473–8484. https://doi.org/10.1021/acsnano.0c02712.

(2) Yang, J.; Zhu, C.; Deng, Y.; Tang, B.; Liu, Z. Magnetism of Two-Dimensional Chromium Tellurides. *iScience* **2023**, *26* (5), 106567. https://doi.org/10.1016/j.isci.2023.106567.
